# K-stability of Fano 3-folds in the World of Null-A

**DOI:** 10.1007/s00031-026-09977-9

**Published:** 2026-07-08

**Authors:** Hamid Abban, Ivan Cheltsov, Takashi Kishimoto, Frédéric Mangolte

**Affiliations:** 1 University of Nottingham, Nottingham, England; 2 University of Edinburgh, Edinburgh, Scotland; 3 Saitama University, Saitama, Japan; 4 Aix Marseille University, CNRS, I2M, Marseille, France

## Abstract

A variety is said to satisfy Condition $(\textbf{A})$ if every finite abelian subgroup of its automorphism group has a fixed point. We show that a smooth Fano 3-fold not satisfying Condition $(\textbf{A})$ is K-polystable unless it is contained in eight exceptional deformation families (seven of them consist of one smooth member, and one of them has two-parameter moduli).

## Introduction

The key to understanding the world is to see beyond the apparent and recognize the underlying patterns and connections. ——————————————————————– *The World of Null-A*, A. E. van Vogt, 1948

The study of Fano varieties in algebraic geometry offers a rich interplay between geometric structure, arithmetic properties, and birational classification. They are characterized by their ample anticanonical divisors, a property that endows them with a wealth of positivity and connects them to fundamental questions in the minimal model program and moduli theory. Among Fano varieties, smooth Fano 3-folds hold a privileged position due to their comprehensive classification into 105 deformation families, a monumental achievement by Iskovskikh, Mori, and Mukai. This classification provides a detailed roadmap for exploring their properties, from their Picard groups to their automorphism groups, making them an ideal testing ground for conjectures and theories in modern geometry.

In recent years, the concept of K-polystability has emerged as a transformative invariant in the study of Fano varieties. Rooted in the work of Tian, Donaldson, and others, K-polystability serves as a bridge between algebraic geometry and differential geometry, determining the existence of Kähler-Einstein metrics on Fano manifolds. Beyond its metric implications, K-polystability has profound connections to the construction of moduli spaces, offering a stability condition that governs the compactification of families of Fano varieties. This development has spurred a flurry of research, uncovering surprising links between K-polystability and other invariants, such as the existence of rational points in arithmetic settings or the behavior of automorphism groups in the geometric setting. Our paper builds on this momentum, seeking to deepen these connections by exploring a purely geometric analogue to an arithmetic phenomenon previously identified in the context of smooth Fano 3-folds.

The inspiration for this work stems from a recent result established in prior research [1], which examined smooth Fano 3-folds defined over subfields $\Bbbk \subset \mathbb {C}$. That study revealed that if the geometric model of such a 3-fold is not K-polystable, then the variety typically possesses a k-rational point, barring a small set of exceptional cases. This finding suggested an intimate relationship between K-polystability and the arithmetic property of “pointlessness” (the absence of rational points), raising the question of whether a similar principle might hold in a geometric framework, independent of field considerations. To pursue this, we shift our focus from rational points to the action of finite abelian groups on the variety, introducing a new geometric condition that mirrors the arithmetic one.

Following [21], we define Condition $\mathbf {(A)}$ for a smooth variety *X* as follows: *X* satisfies Condition $\mathbf {(A)}$ if every finite abelian subgroup of its automorphism group ${\text {Aut}}(X)$ fixes at least one point in *X*. This definition draws inspiration from the Lang–Nishimura theorem and Kollár–Szabó’s birational invariance results, which highlight the significance of fixed points in equivariant geometry. Our primary objective is to classify non-K-polystable smooth Fano 3-folds according to whether they satisfy Condition $\mathbf {(A)}$, and to elucidate the exceptional cases where this condition fails, drawing parallels to the arithmetic exceptions identified in [1].

### Main Theorem

Every smooth Fano 3-fold that is not K-polystable satisfies Condition $\mathbf {(A)}$, with exactly eight exceptions. These exceptional varieties are:

- $( \text {N}^{\underline{\text {o}}}{3.28)}$ $\mathbb {P}^1 \times \mathbb {F}_1$, where $\mathbb {F}_1$ is the first Hirzebruch surface.
- $( \text {N}^{\underline{\text {o}}}{4.10)}$ $\mathbb {P}^1 \times S_7$, where $S_7$ is a smooth del Pezzo surface of degree 7.
- $( \text {N}^{\underline{\text {o}}}{2.23)}$ The blowup of a smooth quadric 3-fold $Q\subset \mathbb {P}^4$ along a quartic elliptic curve $C_4\subset Q$ such that the unique hyperplane section of *Q* containing $C_4$ is smooth.
- $( \text {N}^{\underline{\text {o}}}{3.31)}$ The blowup of a quadric cone in $\mathbb {P}^4$ at its vertex.
- $( \text {N}^{\underline{\text {o}}}{4.8)}$ The blowup of $\mathbb {P}^1 \times \mathbb {P}^1 \times \mathbb {P}^1$ along a smooth curve of degree (0, 1, 1).
- $( \text {N}^{\underline{\text {o}}}{2.33)}$ The blowup of $\mathbb {P}^3$ along a line.
- $( \text {N}^{\underline{\text {o}}}{4.12)}$ The blowup of $\mathbb {P}^3$ at two distinct points followed by the blowup of the strict transform of the line through these points.
- $( \text {N}^{\underline{\text {o}}}{5.2)}$ The blowup of $\mathbb {P}^3$ at two distinct points followed by the blowup of the strict transforms of two disjoint lines, one passing through the points and another disjoint from it.

In this paper, we follow the Mori–Mukai numbering of the 105 families, written as “Family N^o^*m*.*n*”, in which *m* is the rank of the Picard group of the 3-fold, ranging from 1 to 10, and *n* is simply a list number (we follow the numbering in Fanography).

For each of the exceptions in the Main Theorem, we explicitly construct finite abelian subgroups of their automorphism groups that act without fixed points in Section 5, confirming their failure to satisfy Condition $\mathbf {(A)}$. Moreover, we observe that these varieties are not K-polystable. Note that, up to isomorphism, each exceptional family in the Main Theorem except Family N^o^2.23 contains a unique smooth member. On the other hand, Family N^o^2.23 contains a proper subfamily consisting of smooth Fano 3-folds that are blowups of a smooth quadric 3-fold $Q\subset \mathbb {P}^4$ along a quartic elliptic curve $C_4\subset Q$ such that the unique hyperplane section of *Q* containing $C_4$ is singular. Members of this subfamily do satisfy Condition $\mathbf {(A)}$ by Remark 3.9, while all other smooth members of Family N^o^2.23 do not satisfy Condition $\mathbf {(A)}$, see Example 5.2. The parallels between these geometric exceptions and the arithmetic exceptions from [1] are striking.

A key corollary follows from the Main Theorem: if a smooth Fano 3-fold is strictly K-semistable, then it satisfies Condition $\mathbf {(A)}$, as all such varieties fall outside the exceptional list. Recall that K-stability is stronger than K-polystability, and K-polystability is stronger than K-semistability, and all exceptional cases in the Main Theorem above are K-unstable (that is not-K-semistable).

### Map of the Proof

Recall that smooth Fano 3-folds have been classified into 105 deformation families by Iskovskikh, Mori and Mukai [31]. To achieve our main result, we partition 105 deformation families of smooth Fano 3-folds into the following three sets:

(i) 53 families in which all smooth elements are K-polystable [3, 4, 6, 10, 12–14, 28, 39, 53];
(ii) 27 families where all smooth members are not K-polystable [4, 27];
(iii) 25 families in which only general members are known to be K-polystable [4].

The first 53 deformation families are

N^o^1.1, N^o^1.2, N^o^1.3, N^o^1.4, N^o^1.5, N^o^1.6, N^o^1.7, N^o^1.8, N^o^1.11, N^o^1.12, N^o^1.13, N^o^1.14, N^o^1.15,

N^o^1.16, N^o^1.17, N^o^2.1, N^o^2.2, N^o^2.3, N^o^2.4, N^o^2.6, N^o^2.7, N^o^2.25, N^o^2.8, N^o^2.15, N^o^2.18, N^o^2.19,

N^o^2.27, N^o^2.29, N^o^2.32, N^o^2.34, N^o^3.1, N^o^3.3, N^o^3.4, N^o^3.9, N^o^3.15, N^o^3.17, N^o^3.19, N^o^3.20, N^o^3.25,

N^o^3.27, N^o^4.1, N^o^4.2, N^o^4.3, N^o^4.4, N^o^4.6, N^o^4.7, N^o^5.1, N^o^5.3, N^o^6.1, N^o^7.1, N^o^8.1, N^o^9.1, N^o^10.1.

These families are irrelevant for us — we listed them for completeness of exposition. Similarly, it follows from [27] that every smooth member of the following 26 families is K-unstable:

N^o^2.23, N^o^2.28, N^o^2.30, N^o^2.31, N^o^2.33, N^o^2.35, N^o^2.36, N^o^3.14, N^o^3.16, N^o^3.18, N^o^3.21, N^o^3.22, N^o^3.23,

N^o^3.24, N^o^3.26, N^o^3.28, N^o^3.29, N^o^3.30, N^o^3.31, N^o^4.5, N^o^4.8, N^o^4.9, N^o^4.10, N^o^4.11, N^o^4.12, N^o^5.2.

Furthermore, it was shown in [4] that N^o^2.26 contains exactly two deformation families, where all varieties in each type are isomorphic. One type contains a smooth K-unstable Fano 3-fold, while the other smooth type is strictly K-semistable. In Section 3.2 we show that each element in 19 of these families satisfy Condition $(\textbf{A})$, and describe all smooth members of the Family N^o^2.23 that satisfy Condition $(\textbf{A})$, hence producing all smooth Fano 3-folds appearing in the Main Theorem as exceptional cases. The remaining families are

N^o^1.9, N^o^1.10, N^o^2.5, N^o^2.9, N^o^2.10, N^o^2.11, N^o^2.12, N^o^2.13, N^o^2.14, N^o^2.16, N^o^2.17, N^o^2.20, N^o^2.21,

N^o^2.22, N^o^2.24, N^o^3.2, N^o^3.5, N^o^3.6, N^o^3.7, N^o^3.8, N^o^3.10, N^o^3.11, N^o^3.12, N^o^3.13, N^o^4.13.

Among these we treat Families N^o^1.10, N^o^2.9, N^o^2.11, N^o^2.13, N^o^2.14, N^o^2.17, N^o^2.20, N^o^2.22, N^o^3.8, N^o^3.11 in Section 3.1 by showing that every smooth member satisfies Condition $(\textbf{A})$, and for the remaining 15 families we prove in Section 4 that all smooth members that do not satisfy Condition $(\textbf{A})$ are K-polystable. Note that Families N^o^2.21, N^o^2.24, N^o^3.5, N^o^3.8, N^o^3.10, N^o^3.12, N^o^3.13, N^o^4.13 are known to have non-K-polystable members. Finally, Section 5 contains crucial examples verifying our arguments. In Section 5.1 we provide examples for each of the exceptional cases in the Main Theorem. Finally, in Section 5.2 we provide examples of K-polystable Fano 3-folds not satisfying Condition (A), which confirms varieties studied in Section 4 do exist. This completes the proof of the Main Theorem.

Throughout this paper, all varieties are projective, normal, irreducible, and defined over the field of complex numbers unless otherwise stated.

## Preliminary Results

True intelligence lies not in knowledge alone, but in the ability to apply that knowledge in creative and innovative ways. ——————————————————————– *The World of Null-A*, A. E. van Vogt, 1948

In this section, we collect some technical results that we will use in this article. Most of these results are known to experts, so the reader may skip them and only consult them as they are referred to. We start with the following well-known result by János Kollár and Endre Szabó.

### Theorem 2.1

([48, Proposition A.4]) Let *X* and $X^\prime$ be smooth varieties acted on by a finite abelian group *A*. Suppose that there exists an *A*-equivariant birational map $\psi :X\dasharrow X^\prime$. Then *X* contains a point fixed by *A* if and only if $X^\prime$ contains a point fixed by *A*.

### Corollary 2.2

Let *p* be a point in $\mathbb {P}^n$, and let $\pi :X\rightarrow \mathbb {P}^n$ be the blowup of this point, where n⩾2. Then every finite abelian subgroup in $\textrm{Aut}(X)$ fixes a point in *X*.

### Proof

Since π is $\textrm{Aut}(X)$-equivariant, the required assertion follows from Theorem 2.1. □

### Remark 2.3

If *X* is a (projective) smooth rationally connected variety, then every finite cyclic group acting on *X* has a fixed point by the holomorphic Lefschetz fixed point formula.

The following lemma is a geometric counter-part of [1, Lemma 2.2], and its special cases are well-known to experts [29]. Its proof was communicated to us by Alex Duncan.

### Lemma 2.4

Let *G* be a finite subgroup in $\textrm{PGL}_{n+1}(\mathbb {C})$ such that there is a *G*-invariant hypersurface $X\subset \mathbb {P}^n$ of degree *d*. Suppose that the integers *d* and n+1 are coprime. Then there exists a subgroup $\widetilde{G}\subset \textrm{GL}_{n+1}(\mathbb {C})$ such that $\phi (\widetilde{G})=G\simeq \widetilde{G}$, where $\phi :\textrm{GL}_{n+1}(\mathbb {C})\rightarrow \textrm{PGL}_{n+1}(\mathbb {C})$ is the natural projection.

### Proof

Recall that the restriction of ϕ to the subgroup $\textrm{SL}_{n+1}(\mathbb {C})\subset \textrm{GL}_{n+1}(\mathbb {C})$ gives a surjective homomorphism $\textrm{SL}_{n+1}(\mathbb {C})\rightarrow \textrm{PGL}_{n+1}(\mathbb {C})$ whose kernel consists of scalar matrices and is isomorphic to $\mathbb {Z}/(n+1)\mathbb {Z}$. Let $\widehat{G}$ be the preimage of the group *G* in $\textrm{SL}_{n+1}(\mathbb {C})$ via this epimorphism, and let $f\in \mathbb {C}[x_1,\ldots ,x_{n+1}]$ be the defining polynomial of the hypersurface *X*. Then *f* is a polynomial of degree *d*, and there exists a character $\eta :\widehat{G}\rightarrow \mathbb {C}^*$ such that

$$ g^*(f)=\eta (g)f $$

for every matrix $g\in \widehat{G}$. In particular, if $g=\lambda I_{n+1}\in \widehat{G}$ for some $\lambda \in \mathbb {C}^*$, then $\eta (g)=\lambda ^d$, where $I_{n+1}$ is the identity (n+1)×(n+1) matrix. Let $\textrm{det}:\textrm{GL}_{n+1}(\mathbb {C})\rightarrow \mathbb {C}^*$ be the character that maps every matrix to its determinant. Then $\textrm{det}\big (\lambda I_{n+1}\big )=\lambda ^{n+1}$ for every $\lambda \in \mathbb {C}^*$. Since *d* and n+1 are coprime, we can use appropriate powers of η and $\textrm{det}$ to construct a homomorphism $\theta :\widehat{G}\rightarrow \mathbb {C}^*$ such that $\theta \big (\lambda I_{n+1}\big )=\frac{1}{\lambda }$ for every $\lambda \in \mathbb {C}^*$ such that the scalar matrix $\lambda I_{n+1}$ is contained in $\widehat{G}$. Now, we let $\widetilde{G}$ be the subgroup in $\textrm{GL}_{n+1}(\mathbb {C})$ defined as follows:

$$ \widetilde{G}=\big \{\theta (g)g\ \big \vert \ g\in \widehat{G}\big \}. $$

Then $\widetilde{G}$ is the required subgroup in $\textrm{GL}_{n+1}(\mathbb {C})$. □

### Corollary 2.5

Let *A* be a finite abelian subgroup in $\textrm{PGL}_{n+1}(\mathbb {C})$. Suppose that there is an *A*-invariant irreducible rational curve $C\subset \mathbb {P}^n$ such that $\textrm{deg}(C)$ is odd, and there exists a finite subgroup $\widetilde{A}\subset \textrm{GL}_{n+1}(\mathbb {C})$ such that $\phi (\widetilde{A})=A\simeq \widetilde{A}$, where $\phi :\textrm{GL}_{n+1}(\mathbb {C})\rightarrow \textrm{PGL}_{n+1}(\mathbb {C})$ is the projection. Then *A* fixes a point in *C*.

### Proof

Let $\nu :\widehat{C}\rightarrow C$ be the normalization of the curve *C*. Then ν is *A*-equivariant, and we have $\widehat{C}\simeq \mathbb {P}^1$. On the other hand, since $\widetilde{A}$ is abelian, there is an *A*-invariant hyperplane *H* in $\mathbb {P}^{n}$ that does not contain *C*, so $\nu ^*(H\vert _{C})$ is an *A*-invariant divisor on $\widehat{C}\simeq \mathbb {P}^1$ of degree $\textrm{deg}(C)$. Since $\widehat{C}\simeq \mathbb {P}^1$ and $\nu ^*(H\vert _{C})$ is an effective *A*-invariant divisor on $\widehat{C}$ (i.e., a hypersurface in $\widehat{C}$ of odd degree), we can apply Lemma 2.4 to $\widehat{C}$. Namely, by Lemma 2.4, the action of the abelian group *A* on $\widehat{C}\simeq \mathbb {P}^1$ is given by a two-dimensional representation of the abelian group *A*, which must be reducible (since *A* is abelian), so *A* fixes a point in $\widehat{C}$, so it also fixes a point in *C* as claimed. □

The following lemma is a geometric counter-part of [1, Lemma 3.4].

### Lemma 2.6

Let *S* be a del Pezzo surface, $A\subseteq \textrm{Aut}(S)$ a finite abelian subgroup, and *C* an *A*-invariant irreducible curve in *S*, let $f:\widetilde{S}\rightarrow S$ be the minimal resolution of singularities, let $\widetilde{C}$ be the strict transform on $\widetilde{S}$ of the curve *C*. If $\widetilde{C}^2<0$, then *A* fixes a point in *C*.

### Proof

Observe that the action of the group *A* lifts to $\widetilde{S}$. If $\widetilde{C}^2<0$, then it follows from the adjunction formula that $\widetilde{C}^2=-1$ and $\widetilde{C}\simeq \mathbb {P}^1$, so there exists an *A*-equivariant contraction $\widetilde{S}\rightarrow \overline{S}$ of the curve $\widetilde{C}$ to a smooth *A*-fixed point *P*. Then the curve $\widetilde{C}$ is identified with the projectivization of the Zariski tangent space $V=T_P(\overline{S})$. Since *A* is abelian, *V* splits as a sum of *A*-invariant one-dimensional subspaces, which means that *C* has an *A*-fixed point. □

### Corollary 2.7

Let *S* be a del Pezzo surface, $A\subseteq \textrm{Aut}(S)$ a finite abelian subgroup, and *C* an *A*-invariant irreducible curve in *S*. Suppose that *A* fixes no points in *S*. Then $C^2\geqslant 0$. Moreover, if $C^2=0$ then *C* is contained in the smooth locus of the surface *S*.

### Corollary 2.8

Let *S* be a del Pezzo surface with at worst quotient singularities, let *A* be a finite abelian subgroup in $\textrm{Aut}(S)$, and $\pi :S\rightarrow S^\prime$ an *A*-equivariant birational morphism; in particular, $S^\prime$ is a normal surface. Then *A* fixes a point in *S* if and only if *A* fixes a point in $S^\prime$.

### Proof

Obviously, if *A* fixes a point in *S*, then *A* fixes a point in $S^\prime$. Thus, to complete the proof, we may assume that *A* does not fix points in *S* but *A* fixes a point $p\in S^\prime$ and seek for a contradiction.

First, we observe that $S^\prime$ also has at worst quotient singularities, and $S^\prime$ is also a del Pezzo surface. Applying relative *A*-equivariant Minimal Model Program to *S* over $S^\prime$, we may assume that

$$ \textrm{rk}\big (\textrm{Pic}^A(S)\big )=\textrm{rk}\big (\textrm{Pic}^A(S^\prime )\big )+1, $$

and all π-exceptional irreducible curves are mapped to *p*. Denote these curves by $E_1,\ldots ,E_r$. If r=1, then we get a contradiction by Corollary 2.7, since $E_1^2<0$. Suppose r⩾2 and consider the following *A*-equivariant commutative diagram:where ϖ is a minimal resolution of $p\in S^\prime$, ϕ is the minimal resolution of singularities of singular points of *S* mapped to *p*, and φ is a birational morphism. Then *A* does not fix points in $\widetilde{S}$, so it follows from Theorem 2.1 that *A* also does not fix points in $\widehat{S}$.

Denote by $F_1,\ldots ,F_k$ the irreducible exceptional curves of the morphism ϖ, which are all smooth and isomorphic to $\mathbb {P}^1$. Every two intersecting curves among them meet transversally at one point, and the dual graph of these curves does not contain cycles. All possibilities for the dual graphs of the curves $F_1,\ldots ,F_k$ are given in [30]. Since the dual graph is connected and *A* does not fix points in $\widehat{S}$, it immediately follows from Iliev’s classification [30] that *A* leaves invariant at least one curve among $F_1,\ldots ,F_k$, which also follows directly from Nadel’s [44, Theorem 4.5].

Without loss of generality, we may assume that $F_1$ is *A*-invariant. Let $\widetilde{F}_1$ be the strict transform of this curve on $\widetilde{S}$. Then $\widetilde{F}_1$ is not ϕ-exceptional, since otherwise $\phi (\widetilde{F}_1)$ would be fixed by *A*. Hence, we conclude that $\phi (\widetilde{F}_1)$ is one of the π-exceptional curves $E_1,\ldots ,E_r$. Now, applying Corollary 2.7 to $\phi (\widetilde{F}_1)$, we obtain a contradiction. □

Lemma 2.6 and its corollaries imply the following result.

### Lemma 2.9

Let *S* be a del Pezzo surface with at worst quotient singularities, let *A* be a finite abelian subgroup in $\textrm{Aut}(S)$ such that *A* does not fix points in *S*, let *C* be an irreducible *A*-invariant curve in *S*, and let Δ be an effective $\mathbb {Q}$-divisor on *S* with $\tau C+\Delta \sim _{\mathbb {Q}} -K_{S}$ for some $\tau \in \mathbb {Q}_{>0}$. Then $\tau \leqslant 2$.

### Proof

Suppose that τ>2. First, let us apply *A*-equivariant Minimal Model Program to the surface *S*. If this gives an *A*-equivariant birational morphism $\pi :S\rightarrow S^\prime$, then $S^\prime$ is a del Pezzo surface with at worst quotient singularities such that $\textrm{rk}\big (\textrm{Pic}^A(S)\big )>\textrm{rk}\big (\textrm{Pic}^A(S^\prime )\big )$. Moreover, it follows from Corollary 2.8 that *A* does not fix points in $S^\prime$, and *C* is not π-exceptional by Corollary 2.7, so that $-K_{S^\prime }\sim _{\mathbb {Q}}\tau C^\prime +\Delta ^\prime$, where $C^\prime =\pi (C)$ and $\Delta ^\prime =\pi (\Delta )$. Therefore, replacing *S* with $S^\prime$, we may assume that the *A*-equivariant Minimal Model Program applied to *S* does not give a birational morphism. Then

- either $$\textrm{rk}(\textrm{Pic}^A(S))=1$$;
- or $\textrm{rk}(\textrm{Pic}^A(S))=2$, and there exist two *A*-equivariant conic bundles $\eta _1:S\rightarrow \mathbb {P}^1$ and $\eta _2:S\rightarrow \mathbb {P}^1$ whose general fibers have non-trivial intersection.

In the latter case, we obtain a contradiction by intersecting $\tau C+\Delta \sim _{\mathbb {Q}} -K_S$ with general fibers of the conic bundles $\eta _1$ and $\eta _2$. Indeed, if $F_i$ is a general fiber of $\eta _i$, then

$$ \tau C\cdot F_i\leqslant \big (\tau C+\Delta \big )\cdot F_i=(-K_S)\cdot F_i=2, $$

which implies that $C\cdot F_i=0$, since τ>2 and $\tau C\cdot F_i$ is a non-negative integer, so *C* is contracted by both conic bundles $\eta _1$ and $\eta _2$, which contradicts to $\textrm{rk}(\textrm{Pic}^A(S))=2$. Thus, we have $\textrm{rk}(\textrm{Pic}^A(S))=1$. Increasing τ if necessary, we may assume that Δ=0, so $-K_{S}\sim _{\mathbb {Q}}\tau C$. Then (*S*, *C*) has purely log terminal singularities. This follows from [18, Lemma 2.4.10] as in the proof of [1, Lemma 3.1]. In particular, *C* is smooth [32]. Moreover, applying Kawamata’s subadjunction theorem [32], we see that $C\simeq \mathbb {P}^1$.

Let $f:\widetilde{S}\rightarrow S$ be the minimal resolution of singularities of the surface *S*, and let $\widetilde{C}$ be the strict transform on $\widetilde{S}$ of the curve *C*. Then *f* is *A*-equivariant, *A* does not fix points in $\widetilde{S}$, we have $\widetilde{C}^2\geqslant 0$ by Lemma 2.6, and it follows from Corollary 2.7 that either $C^2>0$ or $C^2=0$ and *C* is contained in the smooth locus of the surface *S*. Moreover, we have $-K_{\widetilde{S}}\sim _{\mathbb {Q}}\tau \widetilde{C}+\widetilde{B}$, where $\widetilde{B}$ is an effective $\mathbb {Q}$-divisor on $\widetilde{S}$ whose support consists of the *f*-exceptional curves. Now, applying *A*-equivariant Minimal Model Program to $\widetilde{S}$, we obtain an *A*-equivariant birational morphism $h:\widetilde{S}\rightarrow \overline{S}$ such that $\overline{S}$ is a smooth surface, and one of the following two cases holds:

- $\textrm{rk}(\textrm{Pic}^A(\overline{S}))=1$ and $\overline{S}$ is a del Pezzo surface;
- $\textrm{rk}(\textrm{Pic}^A(\overline{S}))=2$ and there exists an *A*-equivariant conic bundle $\eta :\overline{S}\rightarrow \mathbb {P}^1$.

Note that $\widetilde{C}$ is not *h*-exceptional, because $\widetilde{C}^2\geqslant 0$. Set $\overline{C}=h(\widetilde{C})$ and $\overline{B}=h(\widetilde{B})$. Then $-K_{\overline{S}}\sim _{\mathbb {Q}}\tau \overline{C}+\overline{B}$. Hence, if $\textrm{rk}(\textrm{Pic}^A(\overline{S}))=1$, then it follows from [8] that $\overline{S}\simeq \mathbb {P}^2$ and $\overline{C}$ is a line in $\overline{S}$, which implies that *A* fixes a point in $\overline{S}$ contradicting Theorem 2.1. Thus, we conclude that $\textrm{rk}(\textrm{Pic}^A(\overline{S}))=2$ and there exists an *A*-equivariant conic bundle $\eta :\overline{S}\rightarrow \mathbb {P}^1$. Then, intersecting the divisor $\tau \overline{C}+\overline{B}$ with a general fiber of the conic bundle η, we see that $\overline{C}$ is a fiber of η. In particular, $\widetilde{C}^2\leqslant \overline{C}^2=0$, so $\widetilde{C}^2=\overline{C}^2=0$. This implies that *h* is an isomorphism in a neighborhood of the curve $\widetilde{C}$, and the complete linear system $|\widetilde{C}|$ gives the composition morphism $\eta \circ h:\widetilde{S}\rightarrow \mathbb {P}^1$. On the other hand, we have $C^2>0$, since $\textrm{rk}(\textrm{Pic}^A(S))=1$. Thus, we see that *S* is singular, and the curve *C* contains some singular points of the surface *S*. In particular, since *A* does not fix points in *C*, we conclude that *C* contains at least two singular points of the surface *S*.

Recall that (*S*, *C*) has purely log terminal singularities. This implies that all these singular points of the surface *S* contained in *C* are cyclic quotient singularities, see, for example, [46, Theorem 2.1.2]. Moreover, since $(K_S+C)\cdot C=(1-\tau )C^2<0$, it follows from the subadjunction formula [32] and [46, Theorem 2.2.4(i)] that *C* contains at most three singular points of the surface *S* (the proof of this fact is the same as the proof of [46, Lemma 6.1.1(i)]). On the other hands, if *C* contains three singular points of the surface *S*, then it follows from Lemma 2.4 that the action of the abelian group *A* on $C\simeq \mathbb {P}^1$ is given by a reducible two-dimensional representation of the group *A*, so *A* fixes a point in *C*, which contradicts our assumption. Hence, we conclude that *C* contains exactly two singular points of the surface *S* forming one *A*-orbit. Let $P_1$ and $P_2$ be the points of this orbit. Then *f*-exceptional curves mapped to the points $P_1$ and $P_2$ form two disjoint Hirzebruch–Jung strings, which are swapped by the action of the group *A*. Since (*S*, *C*) has purely log terminal singularities, $\widetilde{C}$ intersects only the first curves of these strings, which we denote by $E_1$ and $E_2$. Then there exists the following *A*-equivariant commutative diagram:where *g* is the contraction of all *f*-exceptional curves except for the curves $E_1$ and $E_2$, and *q* is a partial resolution of singularities of the surface *S* that contracts the strict transforms of the curves $E_1$ and $E_2$.

Let $\widehat{C}=g(\widetilde{C})$, $\widehat{E}_1=g(E_1)$, $\widehat{E}_2=g(E_2)$. Then $-K_{\widehat{S}}\sim _{\mathbb {Q}}\tau \widehat{C}+a(\widehat{E}_1+\widehat{E}_2)$ for some $a\in \mathbb {Q}_{>0}$. But $\widehat{C}^2=0$, $\widehat{C}\cdot \widehat{E}_1=\widehat{C}\cdot \widehat{E}_2=1$, and $-K_{\widehat{S}}\cdot \widehat{C}=2$ by adjunction formula, which gives a=1, because

$$ 2=-K_{\widehat{S}}\cdot \widehat{C}=\big (\tau \widehat{C}+a(\widehat{E}_1+\widehat{E}_2)\big )\cdot \widehat{C}=a(\widehat{E}_1+\widehat{E}_2)\cdot \widehat{C}=2a. $$

Hence, since $\widehat{E}_1$ and $\widehat{E}_2$ are smooth, we have $-2=\textrm{deg}\big (K_{\widehat{E}_i}\big )\leqslant \big (K_{\widehat{S}}+\widehat{E}_i\big )\cdot \widehat{E}_i=-\tau \widehat{C}\cdot \widehat{E}_i=-\tau <-2$ by subadjunction formula, which is a contradiction. This completes the proof of the lemma. □

Lemma 2.9 implies the following geometric counterpart of [1, Theorem A], which will be used later in the proof of the Main Theorem.

### Theorem 2.10

Let *S* be a del Pezzo surface with quotient singularities, and let *A* be a finite abelian subgroup in $\textrm{Aut}(S)$. Suppose that *A* does not fix points in *S*. Then *S* is K-polystable.

### Proof

Suppose that *S* is not K-polystable. Then it follows from [27, 38, 55] that *S* contains an *A*-invariant irreducible curve *C* with $\beta (C)\leqslant 0$, where

$$ \beta (C)=1-\frac{1}{(-K_S)^2}\int \limits _{0}^{\tau }\textrm{vol}\big (-K_S-uC\big )du $$

in which $\tau =\textrm{sup}\big \{u\in \mathbb {R}_{\geqslant 0}\ \big \vert \ -K_S-uC\ \text {is pseudo-effective}\big \}$ and $\textrm{vol}(D)$ is the volume of a divisor *D*. Note that $\tau \in \mathbb {Q}_{>0}$ and $-K_{S}\sim _{\mathbb {Q}}\tau C+\Delta$ for some effective $\mathbb {Q}$-divisor Δ on the surface *S* whose support does not contain *C*. Then $\tau \leqslant 2$ by Lemma 2.9.

Let $f:\widetilde{S}\rightarrow S$ be the minimal resolution of singularities of the surface *S*, and let $\widetilde{C}$ be the strict transform on $\widetilde{S}$ of the curve *C*. Then *f* is *A*-equivariant, *A* does not fix points in $\widetilde{S}$, we have $\widetilde{C}^2\geqslant 0$ by Lemma 2.6, and it follows from Corollary 2.7 that either $C^2>0$ or $C^2=0$ and *C* is contained in the smooth locus of the surface *S*. Then, since $\tau \leqslant 2$, it follows from [26, Lemma 9.7] that $C^2=0$, and

$$ -K_S\sim _{\mathbb {Q}}2C+aZ $$

for some $a\in \mathbb {Q}_{>0}$ and some *A*-irreducible curve Z⊂S such that $Z^2=0$. In particular, *C* is contained in the smooth locus of the surface *S*. Hence, by Riemann–Roch formula, the linear system |*C*| is a pencil. Moreover, since $C^2=0$ and *C* is irreducible, this pencil is base point free, so it gives a conic bundle $\eta _1:S\rightarrow \mathbb {P}^1$, because $-K_S$ is ample. Since *S* is a Mori Dream Space, the linear system |*nZ*| is base point free for some positive integer *n*. Since $Z^2=0$ and $-K_S$ is ample, the linear system |*nZ*| it also gives a conic bundle $\eta _2:S\rightarrow \mathbb {P}^1$, and *Z* is a union of fibers of this conic bundle. Hence, replacing *Z* if necessary, we may assume that $Z\cap \textrm{Sing}(S)=\varnothing$, and irreducible components of *Z* are smooth fibers of $\eta _2$. Write $Z=Z_1+\cdots +Z_r$, where $Z_1,\ldots ,Z_r$ are irreducible. Then, using adjunction formula, we get $2C\cdot Z_1=\big (2C+aZ\big )\cdot Z_1=-K_S\cdot Z_1=2$, which gives $C\cdot Z_1=1$. Now, taking the product of $\eta _1$ and $\eta _2$, we obtain an isomorphism $S\simeq \mathbb {P}^1\times \mathbb {P}^1$, which is a contradiction, since $\mathbb {P}^1\times \mathbb {P}^1$ is K-polystable. □

The following simple lemma is very useful in our arguments.

### Lemma 2.11

Let *Q* be a smooth quadric 3-fold in $\mathbb {P}^4$, and let *A* be a subgroup in $\textrm{Aut}(Q)$ that is isomorphic to $(\mathbb {Z}/2\mathbb {Z})^2$. Then *A* fixes a point in *Q*.

### Proof

Note that *Q* is the unique smooth Fano 3-fold in Family N^o^1.16, the action of *A* on the quadric *Q* is induced by its action on $\mathbb {P}^4$, and it follows from Lemma 2.4 that the action on $\mathbb {P}^4$ is given by some 5-dimensional representation of the group *A*, which must split as a sum of five 1-dimensional representations. Since the group *A* has exactly four distinct 1-dimensional representations, we see that *A* pointwise fixes a line $\ell \subset \mathbb {P}^4$, so *A* fixes a point in $\ell \cap Q$. □

Let us show how to apply Lemma 2.11 by proving the following (well-known) result.

### Lemma 2.12

Let $V_5$ be the smooth Fano 3-fold with $-K_{V_5}\sim 2H$ and $H^3=5$, where *H* is an ample Cartier divisor on $V_5$. Then *X* satisfies Condition $\mathbf {(A)}$.

### Proof

Note that $V_5$ is the unique smooth Fano 3-fold in Family N^o^1.15. Standard facts about geometry of this 3-fold can be found in [18, 36]. Let *A* be a finite abelian subgroup of $\textrm{Aut}(V_5)$. Suppose that *A* does not fix points in $V_5$. Then *A* is not cyclic by Remark 2.3. On the other hand, we have $\textrm{Aut}(V_5)\simeq \textrm{PGL}_{2}(\mathbb {C})$. Recall from [5] that $\textrm{PGL}_{2}(\mathbb {C})$ contains a unique non-cyclic finite abelian subgroup up to conjugation, and this group is isomorphic to $(\mathbb {Z}/2\mathbb {Z})^2$. Hence, we conclude that $A\simeq (\mathbb {Z}/2\mathbb {Z})^2$.

Recall from [18] that |*H*| gives an embedding $V_5\hookrightarrow \mathbb {P}^6$, so we identify $V_5$ with its image in $\mathbb {P}^6$. Recall from [36] that the Hilbert scheme of lines on $V_5$ is isomorphic to $\mathbb {P}^2$, and the group $\textrm{Aut}(V_5)$ acts faithfully on this $\mathbb {P}^2$ leaving invariant a smooth conic whose points parametrize lines in $V_5$ with normal bundle $\mathcal {O}_{\mathbb {P}^1}(1)\oplus \mathcal {O}_{\mathbb {P}^1}(-1)$. This implies that $V_5$ contains an *A*-invariant line. Then, projecting from this line, we obtain an *A*-equivariant birational map $V_5\dasharrow Q$, where *Q* is a smooth quadric 3-fold. By Lemma 2.11, the group *A* fixes a point in *Q*, so *A* fixes a point in $V_5$ by Theorem 2.1. □

We will also need the following classification based result, which is a direct consequence of the analysis of Mori cone by Matsuki [43].

### Lemma 2.13

Let *X* be a smooth Fano 3-fold, and let *G* be a finite subgroup in $\textrm{Aut}(X)$. Suppose that either $\textrm{Pic}(X)\simeq \mathbb {Z}^2$ and *X* is not contained in Families N^o^2.6, N^o^2.12, N^o^2.21, N^o^2.32, or *X* is contained in Families N^o^3.5, N^o^3.6, N^o^3.7, N^o^3.8, N^o^3.11, N^o^3.12, N^o^3.14, N^o^3.16, N^o^3.18, N^o^3.21, N^o^3.22, N^o^3.23, N^o^3.24, N^o^3.26, N^o^3.29, N^o^3.30, N^o^4.5, N^o^4.9, N^o^4.11. Then every extremal ray of the Mori cone $\overline{\textrm{NE}}(X)$ is *G*-invariant.

### Proof

We present the idea of the proof and explain it in one case. If $\textrm{Pic}(X)\simeq \mathbb {Z}^2$, the required assertion follows from [47, Theorem 1.2]. In every case, the required assertion follows from [43, § III.3]. For example, if *X* is a member of Family N^o^3.6, then there exists a blow up $\pi :X\rightarrow \mathbb {P}^3$ of a disjoint union of a line *L* and a smooth quartic elliptic curve *C*, and we have the following commutative diagram:2.1where $\pi _L$ and $\pi _C$ are blowups of curves *L* and *C*, respectively, $\varpi _L$ and $\varpi _C$ are blowups of the strict transforms of the curves *L* and *C*, respectively, $\phi _L$ is a $\mathbb {P}^2$-bundle, $\phi _C$ is a fibration into quadric surfaces, $\varphi _L$ is a fibration into quintic del Pezzo surfaces, $\varphi _C$ is a fibration into sextic del Pezzo surfaces, the map η is a (standard) conic bundle, $\textrm{pr}_1$ and $\textrm{pr}_2$ are projections to the first and the second factors, respectively. Hence, we see that the Mori cone $\overline{\textrm{NE}(X)}$ is generated by extremal rays contracted by $\varpi _L$, $\varpi _C$ and η, and the group *G* cannot non-trivially permute these extremal rays. Similarly, one can verify the required assertion in the remaining cases using description of the Mori cone $\overline{\textrm{NE}}(X)$ given in [43]. □

We now turn our attention to some certain stability threshold type invariants that enable useful estimations for proving K-polystability. For a more detailed approach, we refer the reader to [2, 4]. Let *X* be a smooth Fano 3-fold, and let *p* be a point in *X*. Recall that

$$ \delta _p(X)=\inf _{\begin{array}{c} E/X\\ p\in C_X(E) \end{array}}\frac{A_{X}(E)}{S_{X}(E)}, $$

where $S_X(E)=\frac{1}{(-K_X)^3}\int \limits _0^\infty \textrm{vol}(-K_X-uE)du$, the entity $A_X(E)$ is the log-discrepancy of *E*, and the infimum is taken over all prime divisors *E* over *X* whose center $C_X(E)$ on *X* contains *p*. Let *S* be an irreducible smooth surface in *X* that contains *p*. Set

$$ \tau =\textrm{sup}\Big \{u\in \mathbb {R}_{\geqslant 0}\ \big \vert \ \text {the divisor } -K_X-uS \text { is pseudo-effective}\Big \}. $$

For $u\in [0,\tau ]$, let *P*(*u*) be the positive part of the Zariski decomposition of the divisor $-K_X-uS$, and let *N*(*u*) be its negative part. Then $S_X(S)=\frac{1}{-K_X^3}\int \limits _{0}^{\tau }\big (P(u)\big )^3du$. For every prime divisor *F* over *S*, following the notation in [2, 4] set

$$\begin{aligned} S\big (W^S_{\bullet ,\bullet };F\big )= &   \frac{3}{(-K_X)^3}\int \limits _0^\tau \big (P(u)\big \vert _S\big )^2\cdot \textrm{ord}_{F}\big (N(u)\big \vert _{S}\big )du\nonumber \\  &   +\frac{3}{(-K_X)^3}\int \limits _0^\tau \int \limits _0^\infty \textrm{vol}\big (P(u)\big \vert _{S}-vF\big )dvdu. \end{aligned}$$

### Theorem 2.14

([2, 4]) For any point p∈S we have

$$ \delta _p(X)\geqslant \min \Bigg \{\frac{1}{S_X(S)},\inf _{\begin{array}{c} F/S\\ p\in C_S(F) \end{array}}\frac{A_S(F)}{S\big (W^S_{\bullet ,\bullet };F\big )}\Bigg \}, $$

where the infimum is taken by all prime divisors over *S* whose center on *S* contains *p*.

If *E* is a prime divisor over *X* such that its center on *X* is a curve in *S*, we have a similar result:

### Theorem 2.15

([2, 4]) Let *E* be a prime divisor over *X* such that $C_X(E)$ is a curve C⊂S. Then

$$ \frac{A_X(E)}{S_X(E)}\geqslant \min \Bigg \{\frac{1}{S_X(S)},\frac{1}{S\big (W^S_{\bullet ,\bullet };C\big )}\Bigg \}. $$

Let us conclude this section by presenting one local result about singularities of pairs from [54], which will be used in the proof of Lemma 4.2. To state the result, we need to recall notations introduced in [54]. Let $\mathbb {R}^n_{+}=\{(a_1,\ldots ,a_n)\in \mathbb {R}^n\ \vert \ a_1>0,\ldots ,a_n>0\}$. For every $\textbf{a}=(a_1,\ldots ,a_n)\in \mathbb {R}^n_{>0}$, let

$$ Q_{\textbf{a}}= \big \{\textbf{x}=(x_1,\ldots ,x_n)\in \mathbb {R}^n\ \big \vert \ x_1\geqslant 0,\ldots ,x_n>0, \textbf{a}\cdot \textbf{x} < 1\big \}. $$

For every positive rational number λ>0, let

$$ \sigma _{n,\lambda }=\min \big \{\Big |(Q_\textbf{a}\cap \mathbb {Z}^n\big |\ \text {such that}\ \textbf{a}\in \mathbb {R}^n_{+}\ \text {and}\ (\lambda ,\lambda ,\ldots ,\lambda )\in Q_\textbf{a}\Big \}, $$

and let

$$ \bar{\sigma }_{n,\lambda } = \min \Big \{\big |(Q_\textbf{a}\cap \mathbb {Z}^n\big |\ \text {such that}\ \textbf{a}\in \mathbb {R}^n_{+}\ \text {and}\ (\lambda ,\lambda ,\ldots ,\lambda )\in \overline{Q_\textbf{a}}\Big \}, $$

Note that $$\sigma _{n,\lambda }\geqslant \bar{\sigma }_{n,\lambda }$$.

### Theorem 2.16

Let *U* be an (affine) variety of dimension *n*, let *p* be a smooth point of the variety *U*, and let *D* be an effective $\mathbb {Q}$-divisor on *U* such that the pair (*U*, *D*) is log canonical outside of the point *p*. For every positive rational number ϵ>0, and let $\mathcal {J}_\epsilon$ be the multiplier ideal sheaf of the log pair (U,(1-ϵ)D), let $\mathcal {L}_\epsilon$ be the subscheme defined by $\mathcal {J}_\epsilon$. Fix $\lambda \in \mathbb {Q}_{>0}$. If $h^0(\mathcal {O}_{\mathcal {L}_{\epsilon }})<\sigma _{n,\lambda }$ for sufficiently small $\epsilon \ll 1$, then

$$ \textrm{lct}_p(U;D)\geqslant \frac{1}{\lambda +1}. $$

Similarly, if $h^0(\mathcal {O}_{\mathcal {L}_{\epsilon }})<\bar{\sigma }_{n,\lambda }$ for sufficiently small $\epsilon \ll 1$, then

$$ \textrm{lct}_p(U;D)>\frac{1}{\lambda +1}. $$

Moreover, there is an absolute constant $c>\frac{1}{\lambda +1}$, such that $\textrm{lct}_p(U;D)\geqslant c$ if $h^0(\mathcal {O}_{\mathcal {L}_{\epsilon }})<\bar{\sigma }_{n,\lambda }$ for all sufficiently small $\epsilon \ll 1$.

### Proof

By [54, Remark 3.1], the first two inequalities for $\textrm{lct}_p(U;D)$ follows from [54, Corollary 3.5] and its proof. The third inequality follows from the fact that the log canonical thresholds are constructible in families, and all ideals $\mathcal {J}_\epsilon$ can be parametrized by some scheme of finite type. □

If *m* is a positive integer, then it follows from [54, Lemma 5.1] that

$$ \sigma _{2,m}\geqslant \frac{4m^2+3m+3}{2}, $$

and it follows from [54, Lemma 5.2] that $\bar{\sigma }_{2,m}\geqslant m(2m+1)$. This gives

### Corollary 2.17

Let *S* be an (affine) surface, let *p* be its smooth point, and let *D* be an effective $\mathbb {Q}$-divisor on *S* such that the pair (*S*, *D*) is log canonical outside of the point *p*. For every positive rational number ϵ>0, and let $\mathcal {J}_\epsilon$ be the multiplier ideal sheaf of the log pair (S,(1-ϵ)D), let $\mathcal {L}_\epsilon$ be the subscheme defined by $\mathcal {J}_\epsilon$. Fix a positive integer *m*. If $h^0(\mathcal {O}_{\mathcal {L}_{\epsilon }})<\frac{4m^2+3m+3}{2}$ for sufficiently small $\epsilon \ll 1$, then

$$ \textrm{lct}_p(S;D)\geqslant \frac{1}{m+1}. $$

Similarly, if $h^0(\mathcal {O}_{\mathcal {L}_{\epsilon }})<m(2m+1)$ for sufficiently small $\epsilon \ll 1$, then

$$ \textrm{lct}_p(S;D)>\frac{1}{m+1}. $$

Moreover, there is an absolute constant $c>\frac{1}{m+1}$, such that $\textrm{lct}_p(S;D)\geqslant c$ if $h^0(\mathcal {O}_{\mathcal {L}_{\epsilon }})<m(2m+1)$ for all sufficiently small $\epsilon \ll 1$.

Letting m=3 in Corollary 2.17, we get

### Corollary 2.18

Let *S* be an (affine) surface, let *p* be its smooth point, and let *D* be an effective $\mathbb {Q}$-divisor on *S* such that (*S*, *D*) is log canonical outside of the point *p*. For every positive rational number ϵ>0, and let $\mathcal {J}_\epsilon$ be the multiplier ideal sheaf of the log pair (S,(1-ϵ)D), let $\mathcal {L}_\epsilon$ be the subscheme defined by $\mathcal {J}_\epsilon$. Suppose that $h^0(\mathcal {O}_{\mathcal {L}_{\epsilon }})<21$ for all sufficiently small $\epsilon \ll 1$. Then

$$ \textrm{lct}_p(S;D)\geqslant c>\frac{1}{4}, $$

where *c* is some absolute constant larger that $\frac{1}{4}$.

## Smooth Fano 3-Folds Satisfying Condition $\mathbf {(A)}$

Common sense, do what it will, cannot avoid being surprised occasionally. The object of science is to spare it this emotion and create mental habits which shall be in such close accord with the habits of the world as to secure that nothing shall be unexpected. ——————————————————————————————— Bertrand Russel, quoted in *The World of Null-A*, A. E. van Vogt, 1948

In this section, we prove that every member in several deformation families of smooth Fano 3-folds satisfy Condition $\mathbf {(A)}$. As stated in the introduction, there are 25 families of smooth Fano 3-folds containing K-polystable members that either have a non-K-polystable member or the K-stability picture for all smooth elements is lacking. Among them, we single out 10 families and prove in Section 3.1 that any members in those families satisfies Condition $\mathbf {(A)}$. These are Families N^o^1.10, N^o^2.9, N^o^2.11, N^o^2.13, N^o^2.14, N^o^2.17, N^o^2.20, N^o^2.22, N^o^3.8, N^o^3.11. There are also 27 families of smooth Fano 3-folds where every smooth member is known to be non-K-polystable. In Section 3.2 we show that every smooth member in 19 families (out of 27) always satisfies Condition $\mathbf {(A)}$. These are Families N^o^2.26, N^o^2.28, N^o^2.30, N^o^2.31, N^o^2.35, N^o^2.36, N^o^3.14, N^o^3.16, N^o^3.18, N^o^3.21, N^o^3.22, N^o^3.23, N^o^3.24, N^o^3.26, N^o^3.29, N^o^3.30, N^o^4.5, N^o^4.9, N^o^4.11. This leaves 8 families that contain exceptional cases in the Main Theorem, which we deal with in Section 5.1.

### Families Containing K-Polystable Members

#### Lemma 3.1

Let *X* be a smooth Fano 3-fold in Family N^o^1.10. Then *X* satisfies Condition $\mathbf {(A)}$.

#### Proof

Let *A* be a finite abelian group in $\textrm{Aut}(X)$. By Remark 2.3, we may assume that *A* is not cyclic. Recall that $|-K_X|$ is very ample and provides an *A*-equivariant embedding $X\hookrightarrow \mathbb {P}^{13}$ such that the action of *A* on $\mathbb {P}^{13}$ is given by its faithful 14-dimensional representation.

Suppose that *X* contains an *A*-invariant smooth conic *C*. Let $\phi :\widetilde{X}\rightarrow X$ be the blowup along *C*, and let *E* be the ϕ-exceptional surface. Then $(-K_{\widetilde{X}})^3=16$, and it follows from [31, Corollary 4.4.3] that the anticanonical divisor $-K_{\widetilde{X}}$ is nef, and $(-K_{\widetilde{X}})^2\cdot S>0$ for every surface $S\subset \widetilde{X}$. Thus, it follows from [31, Theorem 4.4.11] that the linear system $|-K_{\widetilde{X}}-E|$ gives an *A*-equivariant birational map $\chi :X\dasharrow Q$, where *Q* is a smooth quadric 3-fold in $\mathbb {P}^{4}$. Moreover, we have an *A*-equivariant commutative diagramsuch that π is the blowup of a smooth rational sextic curve $\Gamma \subset Q$, and ζ is a composition of flops. Observe that *A* acts faithfully on Γ. Since $\Gamma \simeq \mathbb {P}^1$, this gives $A\simeq (\mathbb {Z}/2\mathbb {Z})^2$, so *A* fixes a point in *Q* by Lemma 2.11, and *A* fixes a point in *X* by Theorem 2.1.

Thus, we see that *A* fixes a point if *X* contains an *A*-invariant smooth conic. On the other hand, we know from [36, Lemma 4.3.4] that *A* acts faithfully on the Hilbert scheme of conics in *X*, and this Hilbert scheme is isomorphic to $\mathbb {P}^2$ by [36, Theorem 1.1.1]. Moreover, it follows from [34, 52] that singular conics on *X* are parameterized by a quartic curve in $\mathbb {P}^2$. Thus, it follows from Lemma 2.4 that the action of the group *A* on this $\mathbb {P}^2$ is given by its three-dimensional linear representation, which must split into a sum of one-dimensional representations, because *A* is abelian. Hence, we conclude *A* fixes a point in $\mathbb {P}^2$, so *X* contains a (possibly singular) *A*-invariant conic *C*. If *C* is smooth, we are done. If *C* is singular and reduced, then its singular point is fixed by *A*. Similarly, if C=2L for a line L⊂X, then *A* fixes a point in *L* by Corollary 2.5. □

#### Lemma 3.2

Let *X* be a smooth Fano 3-fold in Family N^o^2.9. Then *X* satisfies Condition $\mathbf {(A)}$.

#### Proof

Let *A* be a finite abelian subgroup in $\textrm{Aut}(X)$. By Lemma 2.13, we have an *A*-equivariant commutative diagramin which π is the blowup of a smooth curve *C* of degree 7 and genus 5, η is a conic bundle with discriminant curve $\Delta \subset \mathbb {P}^2$ of degree 5, and the dashed arrow is given by the two-dimensional linear system of all cubic surfaces containing *C*. Since Δ is *A*-invariant, it follows from Lemma 2.4 that $\mathbb {P}^2$ contains an *A*-invariant line *L*, so $\pi _*(\eta ^*(L))$ is an *A*-invariant cubic surface. Now, applying Lemma 2.4 again, we conclude that there is an *A*-fixed point on $\mathbb {P}^3$, hence a fixed point on *X* by Theorem 2.1. □

#### Lemma 3.3

Let *X* be a smooth Fano 3-fold in Family N^o^2.11. Then *X* satisfies Condition $\mathbf {(A)}$.

#### Proof

Let *A* be a finite abelian subgroup in $\textrm{Aut}(X)$. By Lemma 2.13, there exists an *A*-equivariant birational morphism $\pi :X\rightarrow Y$ such that *Y* is a smooth cubic 3-fold in $\mathbb {P}^4$, and π is a blowup of an *A*-invariant line L⊂Y. Applying Lemma 2.4 and its Corollary 2.5, we see that *A* fixes a point in *L*, so it also fixes a point in *X* by Theorem 2.1. □

#### Lemma 3.4

Let *X* be a smooth Fano 3-fold in Family N^o^2.13. Then *X* satisfies Condition $\mathbf {(A)}$.

#### Proof

It follows from [17] that the group $\textrm{Aut}(X)$ is finite, and it follows from Lemma 2.13 that there exists an $\textrm{Aut}(X)$-equivariant commutative diagramwhere *Q* is a smooth quadric 3-fold in $\mathbb {P}^4$, π is the blowup of a degree six smooth curve C⊂Q with genus two, η is a conic bundle, and the dashed arrow is given by the linear subsystem in $|\mathcal {O}_{Q}(2)|$ consisting of all surfaces that contain *C*. Set $L=\mathcal {O}_{Q}(1)\vert _{C}$. Then we have natural group homomorphisms

$$ \textrm{Aut}(X)\simeq \textrm{Aut}(Q,C)\hookrightarrow \textrm{Aut}(\mathbb {P}^4,C)\simeq \textrm{Aut}(C,[L])\hookrightarrow \textrm{Aut}(C), $$

where $\textrm{Aut}(C,[L])$ is the subgroup in $\textrm{Aut}(C)$ consisting of all automorphisms of the curve *C* that preserve the class of the line bundle *L* in $\textrm{Pic}(C)$. Therefore, in the following we will identify $\textrm{Aut}(X)=\textrm{Aut}(Q,C)$ and $\textrm{Aut}(\mathbb {P}^4,C)=\textrm{Aut}(C,[L])$, and we consider $\textrm{Aut}(X)$ as a subgroup of $\textrm{Aut}(C)$. Note that all possibilities for $\textrm{Aut}(C)$ can be found using the online database [35].

Let *A* be an abelian subgroup in $\textrm{Aut}(Q,C)$. By Theorem 2.1, it is enough to show that *A* fixes a point in *Q*. Suppose that this is not the case. Let us seek for a contradiction. First, *A* is not cyclic by Remark 2.3. Second, it follows from Lemma 2.11 that $A\not \simeq (\mathbb {Z}/2\mathbb {Z})^2$. Third, going through all possibilities for $\textrm{Aut}(C)$ given by [35], we see that $\textrm{Aut}(C)\simeq (\mathbb {Z}/2\mathbb {Z})\rtimes \mathfrak {D}_4$, $A\simeq (\mathbb {Z}/2\mathbb {Z})\times (\mathbb {Z}/6\mathbb {Z})$, the signature of the *A*-action on *C* is [0; 2, 2, 6], and *C* is isomorphic to the following hyperelliptic curve:

3.1 $$\begin{aligned} \{z^2=x^6-y^6\}\subset \mathbb {P}(1_{x},1_{y},3_{z}). \end{aligned}$$

Moreover, it follows from Lemma 2.4 that *L* is *A*-linearizable, it follows from [24, (2.2)] that the subgroup in $\textrm{Pic}(C)$ consisting of all *A*-linearizable line bundles is isomorphic to $\mathbb {Z}$, and it follows from [24, (2.3)] that this subgroup is generated by $K_Z$. Hence, the only *A*-linearizable line bundle in $\textrm{Pic}(C)$ of degree 6 is $3K_Z$. This implies that $L\sim 3K_Z$, and *C* is projectively equivalent to the image of the curve (3.1) embedded into $\mathbb {P}^4$ via the Veronese embedding $\mathbb {P}(1_{x},1_{y},3_{z})\hookrightarrow \mathbb {P}^4$ given by $[x:y:z]\mapsto [x^3:x^2y:xy^2:y^3:z]$. Moreover, since *A* is the unique subgroup in $\textrm{Aut}(C)$ isomorphic to $(\mathbb {Z}/2\mathbb {Z})\times (\mathbb {Z}/6\mathbb {Z})$, we may assume that it acts on (3.1) as follows:

$$\begin{aligned} [x:y:z]&\mapsto [x:y:-z],\\ [x:y:z]&\mapsto [\zeta _6x:y:z], \end{aligned}$$

where $\zeta _6$ is a primitive sixth root of unity. Hence, choosing appropriate homogeneous coordinates $x_1$, $x_2$, $x_3$, $x_4$, $x_5$ on $\mathbb {P}^4$, we may assume that $C=\{x_5^2=x_1^2-x_4^2, x_1x_4=x_2x_3, x_2^2=x_1x_3, x_3^2=x_2x_4\}\subset \mathbb {P}^4$, and the action of the group *A* on $\mathbb {P}^4$ is given by

$$\begin{aligned} [x_1:x_2:x_3:x_4:x_5]&\mapsto [x_1:x_2:x_3:x_4:-x_5],\\ [x_1:x_2:x_3:x_4:x_5]&\mapsto [\zeta _6^3x_1:\zeta _6^2x_2:\zeta _6x_3:x_4:x_5]. \end{aligned}$$

In particular, the (hyperelliptic) involution $[x_1:x_2:x_3:x_4:x_5]\mapsto [x_1:x_2:x_3:x_4:-x_5]$ pointwise fixes a hyperplane in $\mathbb {P}^4$, so *A* fixes a point in *Q*, which is a contradiction. This completes the proof of Lemma 3.4. In fact, one can check that there exists no smooth *A*-invariant quadric in $\mathbb {P}^4$ that contains the curve *C*, which shows that there exists no smooth Fano 3-fold in Family N^o^2.13 that is acted faithfully by the group $(\mathbb {Z}/2\mathbb {Z})\times (\mathbb {Z}/6\mathbb {Z})$. □

#### Lemma 3.5

Let *X* be a smooth Fano 3-fold in one of Families N^o^2.14, N^o^2.20 or N^o^2.22. Then *X* satisfies Condition $\mathbf {(A)}$.

#### Proof

In each case, *X* can be obtained by blowing up the smooth quintic del Pezzo 3-fold $V_5$ described in Lemma 2.12 along a smooth curve. Now, applying Lemmas 2.13 and 2.12 and Theorem 2.1, we obtain the required assertion. □

#### Lemma 3.6

Let *X* be a smooth Fano 3-fold in Family N^o^2.17. Then *X* satisfies Condition $\mathbf {(A)}$.

#### Proof

Let *A* be a finite abelian subgroup in $\textrm{Aut}(X)$. Then, by Lemma 2.13, we have the following *A*-equivariant Sarkisov link:where *Q* is a smooth quadric 3-fold in $\mathbb {P}^4$, π is the blowup of a smooth elliptic curve *C* of degree 5, *f* is a blowup of a smooth elliptic curve *Z* of degree 5, and the dashed arrow is given by the linear system of cubic surfaces containing *Z*. Now, applying Lemma 2.4 to $\mathbb {P}^4$ and *Q*, we see that there exists an *A*-invariant hyperplane section of *Q*. Taking its strict transform on $\mathbb {P}^3$, we obtain an *A*-invariant cubic surface in $\mathbb {P}^3$, so it follows from Lemma 2.4 that *A* fixes a point in $\mathbb {P}^3$, which implies that *A* fixes a point in *X* by Theorem 2.1. □

#### Lemma 3.7

Let *X* be a smooth Fano 3-fold in Family N^o^3.8. Then *X* satisfies Condition $\mathbf {(A)}$.

#### Proof

The 3-fold *X* can be obtained as follows. Consider a smooth divisor $Y\subset \mathbb {P}^2_{x,y,z}\times \mathbb {P}^2_{u,v,z}$ given by

$$ xf_2(u,v,w)+yg_2(u,v,w)+zh_2(u,v,w)=0, $$

where $f_2$, $g_2$, $h_2$ are quadratic polynomials in *u*, *v*, *w*, and a birational morphism $\pi :X\rightarrow Y$ that blows up a smooth fiber *C* of the projection $Y\rightarrow \mathbb {P}^2_{x,y,z}$. Let *A* be a finite abelian subgroup in $\textrm{Aut}(X)$. Then it follows from Lemma 2.13 that π is *A*-invariant.

Let $\textrm{pr}_1:Y\rightarrow \mathbb {P}^2_{x,y,z}$ and $\textrm{pr}_2:Y\rightarrow \mathbb {P}^2_{u,v,w}$ be the projections to the first and the second factors, respectively. Then both $\textrm{pr}_1$ and $\textrm{pr}_2$ are *A*-equivariant by Lemma 2.13, and it follows from Lemma 2.4 that *A* fixes a point $p\in \mathbb {P}^2_{u,v,w}$, since $\textrm{pr}_2(C)$ is an *A*-invariant conic. Let *F* be the fiber of $\textrm{pr}_2$ over *p*. Then *F* is *A*-invariant, and $\textrm{pr}_1(F)$ is an *A*-invariant line, so *A* fixes a point in $\textrm{pr}_1(F)$ by Lemma 2.4. Thus, since $\textrm{pr}_1$ induces an isomorphism $F\simeq \textrm{pr}_1(F)$, we see that *A* fixes a point in *F*. □

#### Lemma 3.8

Let *X* be a smooth Fano 3-fold in Family N^o^3.11. Then *X* satisfies Condition $\mathbf {(A)}$.

#### Proof

Let *p* be a point in $\mathbb {P}^3$, and let $\phi :V_7\rightarrow \mathbb {P}^3$ be the blowup of this point. Then there exists a birational morphism $\pi :X\rightarrow V_7$ that blows up a smooth curve $C\subset V_7$ with ϕ(C) a smooth quartic elliptic curve passing through the point *p*. Now, let *A* be a finite abelian subgroup in $\textrm{Aut}(X)$. Then it follows from Lemma 2.13 that π is *A*-equivariant, and *A* fixes a point in $V_7$ by Corollary 2.2, so *A* also fixes a point in *X* by Theorem 2.1. □

### Families Not Containing K-Polystable Members

#### Remark 3.9

Let *Q* be a smooth quadric 3-fold in $\mathbb {P}^4$, let $C_4$ be a smooth quartic elliptic curve in *Q*, let *S* be the unique hyperplane section of *Q* that contains $C_4$, let *X* be the blowup of *Q* along $C_4$, and let $\widetilde{S}$ be the strict transform on *X* of the surface *S*. Then *X* is a smooth Fano 3-fold in Family N^o^2.23, and $\widetilde{S}$ is $\textrm{Aut}(X)$-invariant. If *S* is singular, then $\widetilde{S}$ has a unique singular point, which must be fixed by the group $\textrm{Aut}(X)$, so, in particular, *X* satisfies Condition $\mathbf {(A)}$. On the other hand, if *S* is smooth, then we will show in Example 5.2 later that *X* does not satisfy Condition $\mathbf {(A)}$.

#### Lemma 3.10

Let *X* be a smooth Fano 3-fold in Family N^o^2.26. Then *X* satisfies Condition $\mathbf {(A)}$.

#### Proof

In this case, *X* can be obtained by blowing up del Pezzo 3-fold $V_5$ described in Lemma 2.12 along a line. By applying Lemmas 2.13 and 2.12 and Theorem 2.1, we obtain the required assertion. □

#### Lemma 3.11

Let *X* be a smooth Fano 3-fold in Families N^o^2.28 or N^o^2.30. Then *X* satisfies Condition $\mathbf {(A)}$.

#### Proof

The 3-fold *X* can be obtained by blowing up $\mathbb {P}^3$ along a smooth plane curve *C* of degree 3 or 2. Let *A* be a finite abelian subgroup in $\textrm{Aut}(X)$. Then, by Lemma 2.13, this blowup is *A*-equivariant. Moreover, the group *A* leaves invariant the plane in $\mathbb {P}^3$ that contains *C*, so *A* fixes a point in $\mathbb {P}^3$ by Lemma 2.4, which implies that *A* fixes a point in *X* by Theorem 2.1. □

#### Lemma 3.12

Let *X* be a smooth Fano 3-fold in Family N^o^2.31. Then *X* satisfies Condition $\mathbf {(A)}$.

#### Proof

Let *A* be a finite abelian subgroup in $\textrm{Aut}(X)$. Then, it follows from Lemma 2.13 that there exists an *A*-equivariant birational morphism $\pi :X\rightarrow Q$ such that *Q* is a smooth quadric 3-fold in $\mathbb {P}^4$, and π is a blowup of a line in *Q*. Let *E* be the π-exceptional surface, then *E* is *A*-invariant and $E\simeq \mathbb {F}_1$. Now, applying Corollary 2.2, we see that *A* fixes a point in *E*, so it fixes a point in *X* by Theorem 2.1. □

#### Lemma 3.13

Let *X* be a smooth Fano 3-fold in Family N^o^2.35. Then *X* satisfies Condition $\mathbf {(A)}$.

#### Proof

The 3-fold *X* is unique and is often called $V_7$ — it can be obtained by blowing up $\mathbb {P}^3$ at a point, so required assertion is a special case of Corollary 2.2. □

#### Lemma 3.14

Let *X* be a smooth Fano 3-fold in Family N^o^2.36. Then *X* satisfies Condition $\mathbf {(A)}$.

#### Proof

In this case, $X\simeq \mathbb {P}( {\mathcal {O}}_{\mathbb {P}^2} \oplus {\mathcal {O}}_{\mathbb {P}^2}(-2))$, and it possesses two extremal contractions: a divisorial contraction $\pi :X \rightarrow \mathbb {P}(1,1,1,2)$ and a $\mathbb {P}^1$-bundle $\eta :X \rightarrow \mathbb {P}^2$. Let *A* be a finite abelian subgroup in $\textrm{Aut}(X)$. Then, by Lemma 2.13, both π and η are *A*-equivariant. Let *E* be the π-exceptional divisor. Then $E\simeq \mathbb {P}^2$, and this isomorphism gives $\mathcal {O}_E(E|_E)\simeq \mathcal {O}_{\mathbb {P}^2}(-2)$. Moreover, since $\mathcal {O}_E(E\vert _{E})$ is isomorphic to the normal bundle of the surface *E* in *X*, we see that this line bundle is *A*-linearizable. Then the line bundle $\mathcal {O}_E(-K_E-E\vert _{E})\simeq \mathcal {O}_{\mathbb {P}^2}(1)$ is also *A*-linearizable, which implies that the *A*-action on $E\simeq \mathbb {P}^2$ is given by a 3-dimensional representation of the group *A*, so *A* fixes a point in *E*. □

#### Lemma 3.15

Let *X* be a smooth Fano 3-fold in one of Families N^o^3.14, N^o^3.16, N^o^3.23, N^o^3.26, N^o^3.29, N^o^3.30 or N^o^4.9. Then *X* satisfies Condition $\mathbf {(A)}$.

#### Proof

Let *p* be a point in $\mathbb {P}^3$, and let $\phi :V_7\rightarrow \mathbb {P}^3$ be the blowup of this point. Then there exists a birational morphism $\pi :X\rightarrow V_7$ that blows up a smooth curve *C*. The curve *C* is irreducible if *X* is contained in Families N^o^3.14, N^o^3.16, N^o^3.23, N^o^3.26, N^o^3.29 or N^o^3.30. On the other hand, if *X* is contained in Family N^o^4.9, then *C* is the strict transform of two skew lines in $\mathbb {P}^3$ such that one of them contains *p*. In every case, it follows from Lemma 2.13 that π is *A*-equivariant for any finite abelian subgroup $A\subset \textrm{Aut}(X)$, so *A* fixes a point in *X* by Theorem 2.1, because *A* fixes a point in $V_7$ by Corollary 2.2. □

#### Lemma 3.16

Let *X* be a smooth Fano 3-fold in Family N^o^3.18. Then *X* satisfies Condition $\mathbf {(A)}$.

#### Proof

The Fano 3-fold *X* can be obtained as a blowup $\pi :X\rightarrow \mathbb {P}^3$ along a disjoint union of a smooth conic *C* and a line *L*. Let *A* be a finite abelian subgroup in $\textrm{Aut}(X)$. Then, by Lemma 2.13, π is *A*-equivariant. In particular, the plane containing *C* is *A*-invariant, so, by Lemma 2.4, *A* fixes a point in $\mathbb {P}^3$, hence *A* also fixes a point in *X* by Theorem 2.1. □

#### Lemma 3.17

Let *X* be a smooth Fano 3-fold in Family N^o^3.21. Then *X* satisfies Condition $\mathbf {(A)}$.

#### Proof

In this case, there exists a blowup $\pi :X\rightarrow \mathbb {P}^1\times \mathbb {P}^2$ of a smooth rational curve *C* of degree (2, 1). Let $\textrm{pr}_1:\mathbb {P}^1\times \mathbb {P}^2\rightarrow \mathbb {P}^1$ and $\textrm{pr}_2:\mathbb {P}^1\times \mathbb {P}^2\rightarrow \mathbb {P}^2$ be projections to the first and second factors, respectively. Then $\textrm{pr}_1\vert _{C}:C\rightarrow \mathbb {P}^1$ is a double cover, and $\textrm{pr}_2(C)$ is a line in $\mathbb {P}^2$.

Let *A* be a finite abelian subgroup in $\textrm{Aut}(X)$. Then it follows from Lemma 2.13, π is *A*-equivariant. Since *A* leaves the line $\textrm{pr}_2(C)$ invariant, it follows from Lemma 2.4 that *A* fixes a point $p\in \textrm{pr}_2(C)$. Since the fiber of the projection $\textrm{pr}_2$ over the point *p* intersects the curve *C* at one point, we see that this point is fixed by *A*, so *A* also fixes a point in *X* by Theorem 2.1. □

#### Lemma 3.18

Let *X* be a smooth Fano 3-fold in Family N^o^3.22. Then *X* satisfies Condition $\mathbf {(A)}$.

#### Proof

Let $\textrm{pr}_1:\mathbb {P}^1\times \mathbb {P}^2\rightarrow \mathbb {P}^1$ be the projection to the first factor, let $H_1$ be its fiber, and let *C* be a smooth conic in $H_1\cong \mathbb {P}^2$. Then there is a blowup $\pi :X\rightarrow \mathbb {P}^1\times \mathbb {P}^2$ along the curve *C*. Let *A* be a finite abelian subgroup in $\textrm{Aut}(X)$. Then it follows from Lemma 2.13 that both π and $\textrm{pr}_1$ are *A*-equivariant, and both $H_1$ and *C* are *A*-invariant. Hence, it follows from Lemma 2.4 that *A* fixes a point in $H_1$, so *A* also fixes a point in *X* by Theorem 2.1. □

#### Lemma 3.19

Let *X* be a smooth Fano 3-fold in Family N^o^3.24. Then *X* satisfies Condition $\mathbf {(A)}$.

#### Proof

Let *A* be a finite abelian subgroup in $\textrm{Aut}(X)$. Then it follows from Lemma 2.13 that there is an *A*-equivariant blowup $\pi :X\rightarrow \mathbb {P}^1\times \mathbb {P}^2$ of a smooth *A*-invariant curve *C* of degree (1, 1).

Let $\textrm{pr}_2:\mathbb {P}^1\times \mathbb {P}^2\rightarrow \mathbb {P}^2$ be the projection to the second factor. Then $\textrm{pr}_2$ is *A*-equivariant, and $\textrm{pr}_2(C)$ is an *A*-invariant line in $\mathbb {P}^2$, and it follows from Lemma 2.4 that *A* fixes a point in $\textrm{pr}_2(C)$. Then *A* fixes a point in *C*, since $\textrm{pr}_2|_C:C \rightarrow \textrm{pr}_2(C)$ is an isomorphism, so *A* fixes a point in *X* by Theorem 2.1. □

#### Lemma 3.20

Let *X* be a smooth Fano 3-fold in Family N^o^4.5. Then *X* satisfies Condition $\mathbf {(A)}$.

#### Proof

In this case, there exists a birational morphism $\pi :X\rightarrow \mathbb {P}^1\times \mathbb {P}^2$ that blows up two smooth disjoint curves *C* and *L* such that *C* is a curve of degree (2, 1), and *L* is a curve of degree (1, 0). Let *A* be a finite abelian subgroup in $\textrm{Aut}(X)$. Then it follows from Lemma 2.13 that π is *A*-equivariant, and both curves *C* and *L* are *A*-invariant. Thus, blowing up $\mathbb {P}^1\times \mathbb {P}^2$ along *C* gives us an *A*-equivariant birational morphism to the smooth Fano 3-folds in Family N^o^3.21, and *A* fixes a point in this 3-fold by Lemma 3.17, so *A* also fixes a point in *X* by Theorem 2.1. □

#### Lemma 3.21

Let *X* be a smooth Fano 3-fold in Family N^o^4.11. Then *X* satisfies Condition $\mathbf {(A)}$.

#### Proof

Let $V=\mathbb {P}^1 \times \mathbb {F}_1$, let *S* be a fiber of the natural projection $V\rightarrow \mathbb {P}^1$, and let *C* be the (-1)-curve in $S\cong \mathbb {F}_1$. Then there exists a birational morphism $\pi :X \rightarrow V$ that is the blowup of the curve *C*. Let *A* be a finite abelian subgroup in $\textrm{Aut}(X)$. Then it follows from Lemma 2.13 that π is *A*-equivariant, and *S* is *A*-invariant, so *A* fixes a point in *S* by Corollary 2.2. Hence *A* fixes a point in *X* also by Theorem 2.1. □

## K-Polystable Fano 3-Folds Not Satisfying Condition $\mathbf {(A)}$

It was a pleasure to remember the crystal-like clarity of the man’s writing, the careful. semantic consideration given to every multi- ordinal word used, the breadth of intellect and understanding of the human body-and-mind-as-a-whole. ———————————————————————————– *The World of Null-A*, A. E. van Vogt, 1948

In this section, we work through the 15 families of Fano 3-folds that contain K-polystable elements but K-polystability is not known or does not hold for all elements. These are Families N^o^1.9, N^o^2.5, N^o^2.10, N^o^2.12, N^o^2.16, N^o^2.21, N^o^2.24, N^o^3.2, N^o^3.5, N^o^3.6, N^o^3.7, N^o^3.10, N^o^3.12, N^o^3.13, N^o^4.13. They also exhibit the phenomenon that their smooth elements do not always satisfies Condition $\mathbf {(A)}$. We will constantly use the following lemma.

### Lemma 4.1

Let *X* be a smooth Fano 3-fold, and *G* be a finite subgroup in $\textrm{Aut}(X)$. Suppose that *X* is not K-polystable. Then there exists a *G*-invariant prime divisor $\textbf{F}$ over *X* such that

$$ 1\geqslant \delta (X)=\frac{A_X(\textbf{F})}{S_X(\textbf{F})}. $$

### Proof

It follows from the valuative criterion for K-stability [27, 38] that $\delta (X)\leqslant 1$. Since $\delta (X)<\frac{4}{3}$, it follows from [40, Theorem 1.2] that there exists a prime divisor $\textbf{F}$ over *X* such that

$$ \delta (X)=\frac{A_X(\textbf{F})}{S_X(\textbf{F})}. $$

Moreover, if δ(X)<1, then it follows from [55, Theorem 4.4] that we may choose $\textbf{F}$ to be *G*-invariant. Similarly, if δ(X)=1, then we can also assume that $\textbf{F}$ is *G*-invariant by [55, Corollary 4.14], because *X* is not K-polystable. □

In the arguments in this section, we will use Lemma 4.1 as follows. In each case, we denote by *X* the smooth Fano 3-fold which does not satisfy Condition $\mathbf {(A)}$, and we denote by *A* a finite abelian subgroup in $\textrm{Aut}(X)$ that does not fix points on *X*. We aim to prove that *X* is K-polystable. We assume that *X* is not K-polystable. Then, by Lemma 4.1, there exists an *A*-invariant prime divisor $\textbf{F}$ over *X* such that

$$ 1\geqslant \delta (X)=\frac{A_X(\textbf{F})}{S_X(\textbf{F})}. $$

Let Z⊂X be the center of the divisor $\textbf{F}$. Then, in each case under consideration, *Z* is not a surface by [4, Theorem 3.17]. On the other hand, since *A* does not fix points on *X*, we conclude that *Z* is an irreducible curve (it is irreducible because it is a center of the prime divisor $\textbf{F}$), and $\delta _p(X)\leqslant 1$ for every point p∈Z. Then, we will seek for a contradiction, often by showing that $\delta _p(X)>1$ for a sufficiently general point p∈Z.

### Lemma 4.2

Let *X* be a smooth Fano 3-fold in Family N^o^1.9, and let *A* be a finite abelian subgroup in $\textrm{Aut}(X)$ that does not fix points on *X*. Then *X* is K-polystable.

### Proof

Observe that the linear system $|-K_X|$ gives an *A*-equivariant embedding $X\hookrightarrow \mathbb {P}^{11}$ such that the action of the group *A* on $\mathbb {P}^{11}$ is induced by its faithful 12-dimensional representation. In the following, we identify *X* with its anticanonical image in $\mathbb {P}^{11}$.

Suppose that *X* is not is K-polystable. Let $\textbf{F}$ be an *A*-invariant prime divisor whose existence is asserted by Lemma 4.1, and let *Z* be its center on *X*. Then, as we explained above, *Z* is an irreducible *A*-invariant curve. Note that *Z* is not a line, since otherwise *Z* would contain *A*-fixed point by Lemma 2.4. Moreover, arguing as in the proofs of [19, Proposition 7.5] and [4, Theorem 1.51] and using Nadel vanishing theorem, we see that *Z* is a smooth rational curve of degree 2, 4 or 6, which is projectively normal. In particular, the action of the group *A* on the curve *Z* induces a group homomorphism $A\rightarrow \textrm{Aut}(Z)\simeq \textrm{PGL}_2(\mathbb {C})$ whose image must be $(\mathbb {Z}/2\mathbb {Z})^2$, because *A* does not fix points in *Z*, and $(\mathbb {Z}/2\mathbb {Z})^2$ is the only finite abelian subgroup in $\textrm{Aut}(Z)$ that does not fix points in *A* by [5]. We believe that one can use this to obtain a contradiction. However, we decided to use a more sophisticated approach, which is used in the end of the proof of [3, Theorem 5.12]. Our proof is almost identical to what is done there.

Let *S* be a very general surface in $|-K_X|$, let $H_S=-K_X\vert _{S}$, and let *C* be a general curve in $|H_S|$. Then $\textrm{Pic}(S)=\mathbb {Z}[H_S]$, and $C\cap (S\cap Z)=\varnothing$. Let $V_{\vec {\bullet }}$ be the $\mathbb {Z}_{\geqslant 0}$-graded linear series associated to $-K_X$, let $W_{\vec {\bullet }}$ be its refinement by *S* (see [2, Example 2.1]), and let $\mathcal {F}$ be the filtration induced by *C*. Then it follows from [2, Theorem 3.4] that

$$ \delta _{S\cap Z}(S;W_{\vec {\bullet }},\mathcal {F})\leqslant 1. $$

Let us estimate $\textrm{lct}_{S\cap Z}(S;D)$ for an *m*-basis type divisor *D* of the refinement $W_{\vec {\bullet }}$ compatible with $\mathcal {F}$ for sufficiently large m≫0. If we show that $\textrm{lct}_{S\cap Z}(S;D)$ is greater than a fixed number >1 for all sufficiently large *m*, this would contradict the inequality $\delta _{S\cap Z}(S;W_{\vec {\bullet }},\mathcal {F})\leqslant 1$.^1^

Suppose that *m* is sufficiently large. By [2, § 3.1], there exists an effective $\mathbb {Q}$-divisor Γ such that

$$ D=S_m(W_{\vec {\bullet }};C)C+\Gamma . $$

Since $C\cap (S\cap Z)=\varnothing$, we have $\textrm{lct}_{S\cap Z}(S;D)=\textrm{lct}_{S\cap Z}(S;\Gamma )$. Note that $\Gamma \sim _\mathbb {Q}\lambda _mH_S$ for some $\lambda _m\in \mathbb {Q}_{>0}$, since $\textrm{Pic}(S)=\mathbb {Z}[H_S]$. On the other hand, it follows from [2, (3.1)] that

$$ c_1(W_{\vec {\bullet }})=\big (c_1(V_{\vec {\bullet }})-S(V_{\vec {\bullet }};S)S\big )|_S\sim _{\mathbb {Q}}\frac{3}{4}H_S. $$

Hence, by [2, Lemma 2.13] and the calculation above we get

$$ \lim \limits _{m\rightarrow \infty }S_m(W_{\vec {\bullet }};C)=S(W_{\vec {\bullet }};C)=\frac{3}{4}S(H_S;C)=\frac{1}{4}, $$

because $C\sim H_S$. Therefore, we have $\lambda _m=\frac{3}{4}-S_m(W_{\vec {\bullet }};C)$, because

$$ \Gamma \sim _\mathbb {Q}D-S_m(W_{\vec {\bullet }};C)C=\frac{3}{4}H_S-S_m(W_{\vec {\bullet }};C)H_S. $$

In particular, we have $\lim \limits _{m\rightarrow \infty }\lambda _m=\frac{1}{2}$. On the other hand, it follows from the proof of [2, Lemma 2.9] that there exists a sequence of numbers $r_m\in (0,1)$ with $\lim \limits _{m\rightarrow \infty }r_m=1$ such that

$$ r_mS_m(W_{\vec {\bullet }};E)<S(W_{\vec {\bullet }};E) $$

for any prime divisor *E* over *S*, and the numbers $r_m$ are independent of the choice of *E*. Moreover, the sequence can be slightly perturbed so that we also have $r_m\lambda _m<\frac{1}{2}$. Then the log pair $(S,4r_m\Gamma )$ has Kawamata log terminal singularities away from finitely many points, because

$$ \textrm{ord}_E(r_m\Gamma )\leqslant r_m S_m(W_{\vec {\bullet }};E)<S(W_{\vec {\bullet }};E)=\frac{3}{4}S(H_S;E)\leqslant \frac{1}{4} $$

for every prime divisor E⊂S. Let $\mathcal {J}=\mathcal {J}(S,4r_m\Gamma )$ be the multiplier ideal sheaf of the log pair $(S,4r_m\Gamma )$. Then $\mathcal {J}$ is co-supported at finitely many points, and it follows from Nadel vanishing theorem that

$$ H^1\big (S,\mathcal {J}\otimes \mathcal {O}_S(2H_S)\big )=0, $$

because $2H_S-(K_S+4r_m\Gamma )$ is ample. Hence, applying Riemann–Roch theorem, we get

$$ \ell (\mathcal {O}_S/\mathcal {J})\leqslant h^0\big (S,\mathcal {O}_S(2H_S)\big )=2(H_S^2)+2=38. $$

On the other hand, the intersection S∩Z consists of at least two points, because *Z* is not a line. Therefore, there exists a point $p\in S\cap Z$ such that

$$ \ell (\mathcal {O}_{S,p}/\mathcal {J}_p)\leqslant 19\leqslant 21. $$

So, it follows from Corollary 2.18 that $\textrm{lct}_p(S,4r_m\Gamma )\geqslant c$ for some absolute constant $c>\frac{1}{4}$. This gives

$$ \textrm{lct}_{S\cap Z}(S;D)=\textrm{lct}_{S\cap Z}(S,\Gamma )\geqslant \textrm{lct}_p(S,\Gamma )\geqslant 4r_mc. $$

Since $\lim \limits _{m\rightarrow \infty }r_m=1$ and $c>\frac{1}{4}$, we conclude that $\textrm{lct}_{S\cap Z}(S,D)$ is greater than a fixed number >1 for all sufficiently large *m*, which contradicts the inequality $\delta _{S\cap Z}(S;W_{\vec {\bullet }},\mathcal {F})\leqslant 1$ obtained earlier. □

### Lemma 4.3

Let *X* be a smooth Fano 3-fold in Families N^o^2.5, N^o^2.10 or N^o^3.7, and let *A* be a finite abelian subgroup in $\textrm{Aut}(X)$ that does not fix points on *X*. Then *X* is K-polystable.

### Proof

Following [43] and unifying the three cases, in the following diagram4.1*V* is one of the following 3-folds: if *X* is contained in Family N^o^2.5 then *V* is a smooth cubic 3-fold in $\mathbb {P}^4$ (belonging to Family N^o^1.13), if *X* is contained in Family N^o^2.10 then*V* is a smooth complete intersection of two quadrics in $\mathbb {P}^5$ (belonging to Family N^o^1.14), and if *X* is contained in Family N^o^3.7 then *V* is a smooth divisor of degree (1, 1) in $\mathbb {P}^2\times \mathbb {P}^2$ (belonging to Family N^o^2.32). The morphism π is the blowup of an *A*-invariant smooth elliptic curve C⊂V, and ϕ is a fibration into del Pezzo surfaces of degree *d*, where

$$ d={\left\{ \begin{array}{ll} & 3\ \text {if } X \text { contained in Family } \text {N}^{\underline{\text {o}}} 2.5 , \\ & 4\ \text {if } X \text { contained in Family } \text {N}^{\underline{\text {o}}} 2.10 , \\ & 6\ \text {if } X \text { contained in Family } \text {N}^{\underline{\text {o}}} 3.7 . \end{array}\right. } $$

Note that every fiber of ϕ is irreducible, reduced and normal. Indeed, if *F* is a fiber of ϕ, then ϕ(F) is a surface in *V* that is smooth along the curve *C*, because otherwise *C* would be singular, since *C* is a complete intersection of ϕ(F) add another surface in the linear system |ϕ(F)|. In particular, we see that $F\simeq \phi (F)$, and ϕ(F) has isolated singularities, because *C* is cut out by an ample divisor on ϕ(F). This implies that *F* is irreducible, reduced and normal. Moreover, if *X* is contained in Family N^o^2.10 or in Family N^o^3.7, then all fibers of ϕ have at worst Du Val singularities. Furthermore, in each case (4.1) is *A*-equivariant by Lemma 2.13.

Suppose that *X* is not K-polystable. Let $\textbf{F}$ be an *A*-invariant divisor whose existence is asserted by Lemma 4.1, and let *Z* be its center on *X*. Then, as explained above, *Z* is an irreducible *A*-invariant curve.

Let *E* be the π-exceptional surface. Then the natural projection E→C and the restriction $\phi \vert _{E}:E\rightarrow \mathbb {P}^1$ gives an isomorphism $E\simeq C\times \mathbb {P}^1$, because every fiber of the restriction $\phi \vert _{E}:E\rightarrow \mathbb {P}^1$ is a section of the natural projection E→C. We claim that $Z\not \subset E$. Indeed, suppose that Z⊂E. Then π(Z)=C, since otherwise π(Z) would be an *A*-fixed point which is impossible by Theorem 2.1. Let *H* be the class of a divisor in $\textrm{Pic}(V)$ such that $-K_{V}\sim 2H$, and let *u* be a non-negative real number. Then

$$ -K_{X}-uE\sim _{\mathbb {R}}\pi ^*(2H)-(1+u)E, $$

which implies that the divisor $-K_{X}-uE$ is pseudoeffective if and only if u⩽1, and for every u∈[0,1], the divisor $-K_{X}-uE$ is nef. This gives $S_{X}(E)=\frac{1}{4d}\int \limits _{0}^{1}\big (-K_{X}-uE\big )^3du=\frac{1}{4d}\int \limits _{0}^{1}d(2u^3-6u+4)du=\frac{3}{8}$. Then we have $S(W_{\bullet ,\bullet }^{E}; Z)\geqslant 1$ by Theorem 2.15, where $S\big (W_{\bullet ,\bullet }^{E}; Z\big )=\frac{3}{4d}\int \limits _{0}^{1}\int \limits _0^\infty \textrm{vol}\big ((-K_{X}-uE)\vert _{S}-vZ\big )dvdu$. Let $\textbf{s}$ be a fiber of the restriction $\phi \vert _{E}:E\rightarrow \mathbb {P}^1$, and let $\textbf{f}$ be a fiber of the natural projection E→C. Then $Z\equiv a\textbf{s}+b\textbf{f}$ for some non-negative integers *a* and *b*, where a⩾1, since π(Z)=C. This gives

$$\begin{aligned} 1\leqslant &   S\big (W_{\bullet ,\bullet }^{E}; Z\big ) = \frac{3}{4d}\int \limits _{0}^{1}\int \limits _0^\infty \textrm{vol}\Big (\big (-K_{X}-uE\big )\big \vert _{S}-v(a\textbf{s}+b\textbf{f})\Big )dvdu\nonumber \\\leqslant &   \frac{3}{4d}\int \limits _{0}^{1}\int \limits _0^\infty \textrm{vol}\Big (\big (-K_{X}-uE\big )\big \vert _{S}-v\textbf{s}\Big )dvdu=\\= &   \frac{3}{4d}\int \limits _{0}^{1}\int \limits _{0}^{1+u}\big ((1+u-v)\textbf{s}+d(1-u)\textbf{f}\big )^2dvdu\\= &   \frac{3}{4d} \int \limits _{0}^{1} \int \limits _{0}^{1+u} 2d(1-u)(1+u-v)dvdu=\frac{11}{16}, \end{aligned}$$

which is absurd. Hence, we conclude that $Z\not \subset E$.

Let *p* be sufficiently general point in *Z*, and let *S* be the fiber of ϕ that contains *p*. Then

$$ 1\geqslant \delta (X)=\frac{A_X(\textbf{F})}{S_X(\textbf{F})}\geqslant \delta _p(X), $$

because p∈Z. Note that $p\not \in E$, since $Z\not \subset E$. Thus, if *S* has Du Val singularities, then

4.2 $$\begin{aligned} 1\geqslant \delta _p(X)\geqslant \textrm{min}\Big \{\frac{16}{11},\frac{16}{15}\delta (S)\Big \}. \end{aligned}$$

Indeed, if *X* is contained in Family N^o^2.5, this follows from [10, Lemma 2.1]. Meanwhile, even if *X* is in the Family N^o^2.10 or N^o^3.7, then the similar argument as in the proof of [10, Lemma 2.1] can be applicable to obtain (4.2). To be more precise, for the Family N^o^2.10 (resp. N^o^3.7), we have only to substitute d=4 (resp. d=6) in the proof of [10, Lemma 2.1]. Hence, if *S* has Du Val singularities, then (4.2) gives δ(S)<1, so *S* is K-unstable. On the other hand, if $\phi (Z)=\mathbb {P}^1$, then the surface *S* is smooth and it is known to be K-polystable [4]. Hence, we conclude that ϕ(Z) is a point in $\mathbb {P}^1$, so Z⊂S, which implies that *S* is *A*-invariant. Moreover, if *S* has Du Val singularities, then the surface *S* is K-polystable by Theorem 2.10, which is a contradiction. Hence, we see that *X* is contained in Family N^o^2.5 (because all fibers of ϕ admit at most Du Val singularities as remarked), and *S* is a cubic cone, so its vertex is fixed by *A*, which is a contradiction. □

### Lemma 4.4

Let *X* be a smooth Fano 3-fold in Family N^o^2.12, and let *A* be a finite abelian subgroup in $\textrm{Aut}(X)$ that does not fix points on *X*. Then *X* is K-polystable.

### Proof

Assume that *X* is not *K*-polystable. Then there exists an *A*-invariant prime divisor ${\textbf{F}}$ over *X* such that $\beta ({\textbf{F}}) \le 0$ and its center *Z* on *X* is a point contained in the intersection of the exceptional divisors of extremal contractions by [15, Theorem A]. Then *Z* is an *A*-fixed point, a contradiction. □

### Lemma 4.5

Let *X* be a smooth Fano 3-fold in Family N^o^2.16, and let *A* be a finite abelian subgroup in $\textrm{Aut}(X)$ that does not fix points on *X*. Then *X* is K-polystable.

### Proof

Using Lemma 2.13, we see that there exists an *A*-equivariant birational morphism f:X→V such that *V* is a smooth complete intersection of two quadrics in $\mathbb {P}^5$, and *f* is a blowup of a conic C⊂V. Moreover, we have the following *A*-equivariant commutative diagram:where π is a conic bundle whose discriminant curve is a (possibly singular) reduced quartic curve $\Delta _4\subset \mathbb {P}^2$, and the dashed arrow is the projection from the plane in $\mathbb {P}^5$ containing *C*. Note that *A* does not fix points in *V* by Theorem 2.1, but it follows from Lemma 2.4 that *A* fixes a point in $\mathbb {P}^2$.

We claim that *A* does not fix points in the curve $\Delta _4$. Indeed, suppose that *A* fixes a point $p\in \Delta _4$. Let *Z* be the fiber of the conic bundle π over *p* with reduced structure. Then *Z* is an *A*-invariant curve. Moreover, if *p* is a smooth point of the curve $\Delta _4$, then *Z* has one singular point, so this point must be fixed by *A*, which contradicts our assumption. Thus, we see that *p* is a singular point of the curve $\Delta _4$. Then *f*(*Z*) is a line in *V* that intersects the conic *C* by one point, so the intersection point f(Z)∩C must be fixed by *A*, which is impossible. Thus, we see that *A* does not fix points in $\Delta _4$.

Let *E* be the *f*-exceptional surface, and let *H* be the preimage on *X* of a sufficiently general hyperplane section of the complete intersection *V*. Then π is given by |H-E|. We claim that $E\simeq \mathbb {P}^1\times \mathbb {P}^1$ or $E\simeq \mathbb {F}_2$. Indeed, the normal bundle of the conic $C\simeq \mathbb {P}^1$ is $\mathcal {O}_{\mathbb {P}^1}(a)\oplus \mathcal {O}_{\mathbb {P}^1}(b)$, where a⩽b and a+b=2. Set n=b-a. Then $E\simeq \mathbb {F}_{n}$. Let $\textbf{l}$ be a fiber of the natural projection E→C, and let $\textbf{s}$ be a section of this projection such that $\textbf{s}^2=-n$. Then $H\vert _{E}\sim 2\textbf{l}$ and $-E\big \vert _{E}\sim \textbf{s}+\frac{n-2}{2}\textbf{l}$. Thus, since $(H-E)\vert _{E}$ is nef, we get

$$ 0\leqslant (H-E)\vert _{E}\cdot \textbf{s}=\frac{2-n}{2}, $$

so n=0 or n=2 as claimed. If n=0, the restriction $\pi \vert _{E}:E\rightarrow \mathbb {P}^2$ is a double cover ramified in a conic. Similarly, if n=2, then $\pi \vert _{E}$ is a contraction of the (-2)-curve $\textbf{s}$ followed by the double cover ramified in a union of two distinct lines.

Now, we are ready to show that *X* is K-polystable. Assume the contrary and let $\textbf{F}$ be an *A*-invariant divisor whose existence is asserted by Lemma 4.1, and let *Z* be its center on *X*. Then, as explained above, *Z* is an *A*-invariant curve in *X*. We claim that $Z\not \subset E$. Indeed, suppose that Z⊂E. Then f(Z)=C, since *A* does not fix points in *C*. Let *u* be a non-negative real number. Then $-K_X-uE$ is pseudoeffective if and only if u⩽1. Moreover, the divisor $-K_X-uE$ is nef for u∈[0,1]. Therefore, integrating $(-K_X-uE)^3=2u^3-6u^2-18u+22$, we obtain $S_X(E)=\frac{23}{44}$. Then, it follows from Theorem 2.15 that

4.3 $$\begin{aligned} 1\geqslant \frac{A_X(\textbf{F})}{S_X(\textbf{F})}\geqslant \min \left\{ \frac{44}{23},\frac{1}{S(W_{\bullet ,\bullet }^E;Z)}\right\} \end{aligned}$$

where $S\big (W_{\bullet ,\bullet }^E; Z\big )=\frac{3}{22}\int \limits _{0}^{1}\int \limits _0^\infty \textrm{vol}\big ((-K_X-uE)\big \vert _{E}-vZ\big )dvdu$. On the other hand, we have

$$ (-K_X-uE)\big \vert _{E}\sim _{\mathbb {R}} {\left\{ \begin{array}{ll} & (1+u)\textbf{s}+(3-u)\textbf{l}\ \text { if } E\cong \mathbb {P}^1\times \mathbb {P}^1 , \\ & (1+u)\textbf{s}+4\textbf{l}\ \text { if } E\cong \mathbb {F}_2 , \end{array}\right. } $$

Hence, if $E\cong \mathbb {P}^1\times \mathbb {P}^1$, then

$$\begin{aligned}\begin{array}{c} S\big (W^E_{\bullet ,\bullet };Z\big )\leqslant \frac{3}{22}\int \limits _0^1\int \limits _0^\infty \textrm{vol}\big ((-K_X-uE)\big \vert _{E}-v\textbf{s}\big )dvdu= \\ =\frac{3}{22}\int \limits _0^1\int \limits _0^{1+u}\big ((1+u-v)\textbf{s}+(3-u)\textbf{l}\big )^2dvdu \!=\! \frac{3}{22}\int \limits _0^1\int \limits _0^{1+u}2(3-u)(1+u-v)dvdu \!=\! \frac{67}{88}, \end{array}\end{aligned}$$

because $|Z-\textbf{s}|\ne \varnothing$, since f(Z)=C. Similarly, if $E\cong \mathbb {F}_2$, we estimate $S(W^E_{\bullet ,\bullet };Z)\leqslant \frac{41}{44}$. In both cases, we have $S(W^E_{\bullet ,\bullet };Z)<1$, which contradicts (4.3). This shows that $Z\not \subset E$ as claimed.

Since *A* does not fix points in $\Delta _4$, we see that π(Z) is not a point in the curve $\Delta _4$. Then

1. either π(Z) is a point in $\mathbb {P}^2$ and *Z* is a smooth fiber of the conic bundle π,
2. or π(Z) is a curve in $\mathbb {P}^2$, which can be an irreducible component of the curve $\Delta _4$.

In both cases, let *p* be a sufficiently general point in *Z*, let $\mathscr {C}$ be the fiber of the conic bundle π that contains *p*, and let *S* be a general surface in |H-E| that contains $\mathscr {C}$. Then $\mathscr {C}$ is reduced, which implies that the surface *S* is smooth. Recall that

$$ 1\geqslant \delta (X)=\frac{A_X(\textbf{F})}{S_X(\textbf{F})}\geqslant \delta _p(X), $$

because p∈Z. Note that $p\not \in E$, since $Z\not \subset E$.

We claim that *S* is a smooth del Pezzo surface of degree 4. Indeed, since$-K_S\sim H\vert _{S}$ and $(-K_S)^2=4$, the divisor $-K_S$ is nef and big, and the only curves that have trivial intersection with it are the fibers of the natural projection E→C contained in *S*. On the other hand, the intersection E∩S is an irreducible curve that is not a fiber of the projection E→C unless $E\simeq \mathbb {F}_2$ and $\mathscr {C}=\textbf{s}$. The latter possibility cannot hold in our case, since $p\in \mathscr {C}$ and $p\not \in E$. Thus, we see that *S* is a smooth del Pezzo surface of degree 4.

Let us apply Theorem 2.14 to *S* to estimate $\delta _p(X)$ from below. Since $-K_X-uS\sim _{\mathbb {R}}(2-u)H-(1-u)E$, the divisor $-K_X-uS$ is pseudoeffective for u∈[0,2], and it is not pseudoeffective for u>2. For u∈[0,2], let *P*(*u*) be the positive part of the Zariski decomposition of the divisor $-K_X-uS$ and let *N*(*u*) be its negative part. Then

$$ P(u)= {\left\{ \begin{array}{ll} & (2-u)H-(1-u)E \ \text { if } 0\leqslant u\leqslant 1 , \\ & (2-u)H\ \text { if } 1\leqslant u\leqslant 2 , \end{array}\right. } $$

and

$$ N(u)= {\left\{ \begin{array}{ll} & 0 \ \text { if } 0\leqslant u\leqslant 1, \\ & (u-1)E\ \text { if } 1\leqslant u\leqslant 2 . \end{array}\right. } $$

Thus, integrating, we get $S_X(S)=\frac{1}{22}\int \limits _{0}^{2}\big (P(u)\big )^3du=\frac{1}{22}\int \limits _{0}^{1}6u^2-24u+22du+\frac{1}{22}\int \limits _{1}^{2}4(2-u)^3du=\frac{13}{22}$, so it follows from Theorem 2.14 that

4.4 $$\begin{aligned} \delta _p(X)\geqslant \min \Bigg \{\frac{22}{13},\inf _{\begin{array}{c} F/S\\ p\in C_S(F) \end{array}}\frac{A_S(F)}{S\big (W^S_{\bullet ,\bullet };F\big )}\Bigg \}, \end{aligned}$$

where $S\big (W^S_{\bullet ,\bullet };F\big )=\frac{3}{22}\int \limits _0^2\int \limits _0^\infty \textrm{vol}\big (P(u)\big \vert _{S}-vF\big )dvdu$, since $p\not \in E$, and the infimum in (4.4) is taken by all prime divisors over *S* whose center on *S* contains *p*. To estimate this infimum, we note that

$$ P(u)\big \vert _{S}\sim _{\mathbb {R}} {\left\{ \begin{array}{ll} & -K_S+(1-u)\mathscr {C} \ \text { if } 0\leqslant u\leqslant 1, \\ & (2-u)(-K_S)\ \text { if } 1\leqslant u\leqslant 2, \end{array}\right. } $$

we set $D_u=-K_S+(1-u)\mathscr {C}$, and we let

$$ \delta _p(S,D_u)=\inf _{\begin{array}{c} F/S\\ p\in C_S(F) \end{array}}\frac{A_{S}(F)}{S_{D_u}(F)}, $$

where the infimum is taken over all prime divisors *F* over the surface *S* whose center on *S* contains *p*, and

$$ S_{D_u}(F)=\frac{1}{D_u^2}\int \limits _0^\infty \textrm{vol}\big (D_u-vF\big )du=\frac{1}{8-4u}\int \limits _0^\infty \textrm{vol}\big (D_u-vF\big )du. $$

Then if follows from [13, Lemma 24] that

4.5 $$\begin{aligned} \delta _p(S,D_u)\geqslant {\left\{ \begin{array}{ll} & \frac{24}{u^2-10u+28}\ \text { if } \mathscr {C} \text { is irreducible}, \\ & \frac{48-24u}{12u^2-54u+61}\ \text { if } \mathscr {C} \text { is reducible}, \end{array}\right. } \end{aligned}$$

and it follows from [4, Lemma 2.12] that $\delta _p(S,D_1)=\delta _p(S)\geqslant \delta (S)\geqslant \frac{4}{3}$. Thus, if $\mathscr {C}$ is irreducible, then

$$\begin{aligned} S\big (W^S_{\bullet ,\bullet };F\big )= &   \frac{3}{22}\int \limits _0^1\int \limits _0^\infty \textrm{vol}\big (-K_S+(1-u)C-vF\big )dvdu \\  &   +\frac{3}{22}\int \limits _1^2\int \limits _0^\infty \textrm{vol}\big ((2-u)(-K_S)-vF\big )dvdu\leqslant \\\leqslant &   \frac{3}{22}A_S(F)\int \limits _0^1\frac{(8-4u)(u^2-10u+28)}{24}du\\  &   +\frac{3}{22}\int \limits _1^2\int \limits _0^\infty \textrm{vol}\big ((2-u)(-K_S)-vF\big )dvdu=\\= &   \frac{13}{16}A_S(F)+\frac{3}{22}\int \limits _1^2\int \limits _0^\infty (2-u)^2\textrm{vol}\big (-K_S-\frac{v}{2-u}F\big )dvdu=\\= &   \frac{13}{16}A_S(F)+\frac{3}{22}\int \limits _1^2(2-u)^3\int \limits _0^\infty \textrm{vol}\big (-K_S-vF\big )dvdu \\\leqslant &   \frac{13}{16}A_S(F)+\frac{3}{22}\int \limits _1^2(2-u)^3\frac{4}{\delta _p(S)}A_S(F)du\leqslant \\\leqslant &   \frac{13}{16}A_S(F)+\frac{3}{22}\int \limits _1^23(2-u)^3A_S(F)du\\= &   \frac{13}{16}A_S(F)+\frac{9}{88}A_S(F)=\frac{161}{176}A_S(F) \end{aligned}$$

for every prime divisor *F* over *S* such that $p\in C_S(F)$, so it follows from (4.4) that $\delta _p(X)\geqslant \frac{176}{161}$ in this case. Similarly, if $\mathscr {C}$ is reducible, get $\delta _p(X)\geqslant \frac{88}{85}$. So, $\delta _p(X)>1$ in both cases, which is a contradiction. □

### Lemma 4.6

Let *X* be a smooth Fano 3-fold in Family N^o^2.21, and let *A* be a finite abelian subgroup in $\textrm{Aut}(X)$ that does not fix points on *X*. Then *X* is K-polystable.

### Proof

The required assertion follows from [41, 42]. Indeed, it follows from [41] that there exists an involution $\tau \in \textrm{Aut}(X)$ such that such that $\textrm{Pic}(X)^{\langle \tau \rangle }=\mathbb {Z}[-K_X]$, and we have the following commutative diagram:where *Q* is a smooth quadric 3-fold in $\mathbb {P}^4$, π is the blowup of a smooth rational twisted quartic curve *C*, and ι is a birational involution given by the linear subsystem in $|\mathcal {O}_Q(2)|$ consisting of surfaces passing through *C*. Moreover, since π is $\textrm{Aut}(Q;C)$-equivariant, we can identify $\textrm{Aut}(Q;C)$ with a subgroup of the group $\textrm{Aut}(X)$. Then we can choose $\tau \in \textrm{Aut}(X)$ such that τ commutes with every element in $\textrm{Aut}(Q;C)$, so, in particular, we have

$$ \textrm{Aut}(X)\simeq \textrm{Aut}(Q;C)\times \mathbb {Z}/2\mathbb {Z}. $$

The paper [41] also lists all possibilities for the group $\textrm{Aut}(Q;C)$. Furthermore, it follows from [42] that *X* is K-pollystable if and only if $\textrm{Aut}(Q;C)$ contains a subgroup isomorphic to $(\mathbb {Z}/2\mathbb {Z})^2$. Now, it is very easy to deduce from [41] that *X* is K-polystable.

Alternatively, we can prove the required assertion using only technical results obtained in [42]. Namely, suppose that *X* is not K-polystable. Let $\textbf{F}$ be an *A*-invariant divisor provided by Lemma 4.1, let *Z* be its center on *X*, and let *p* be a general point in *Z*. Then, as explained above, *Z* is an irreducible *A*-invariant curve, and $\delta _p(X)\leqslant 1$. Then it follows from [42, Technical Theorem 1] that *p* is contained in the union of the exceptional surfaces of the blowups π and $\pi \circ \tau$. Without loss of generality, we may assume that *Z* is contained in the π-exceptional surface. Then it follows from [42, Technical Theorem 2] that π(Z) is a point, which implies that $\pi ^\prime (Z)$ is a line. Hence, the group *A* preserves both extremal rays of the Mori cone $\overline{\textrm{NE}}(X)$, and the diagram above must be *A*-equivariant. Then *A* fixes the point π(Z), which is impossible by Theorem 2.1. □

### Lemma 4.7

Let *X* be a smooth Fano 3-fold in Family N^o^2.24, and let *A* be a finite abelian subgroup in $\textrm{Aut}(X)$ that does not fix points on *X*. Then *X* is K-polystable.

### Proof

Suppose that *X* is not K-polystable. Then it follows from [4, § 4.7] that *X* is a smooth divisor of degree (1, 2) in $\mathbb {P}^2_{x,y,z}\times \mathbb {P}^2_{u,v,w}$ such that either

4.6 $$\begin{aligned} X=\big \{\big (vw+u^2\big )x+\big (uw+v^2\big )y+w^2z=0\big \}, \end{aligned}$$

or

4.7 $$\begin{aligned} X=\big \{\big (vw+u^2\big )x+v^2y+w^2z=0\big \}. \end{aligned}$$

Let $\textrm{pr}_1:X\rightarrow \mathbb {P}^2$ and $\textrm{pr}_2:X\rightarrow \mathbb {P}^2$ be the projections to the first and the second factors, respectively. Then $\textrm{pr}_1$ is an *A* equivariant conic bundle, and $\textrm{pr}_2$ is an *A*-equivariant $\mathbb {P}^1$-bundle. Let $\Delta _3$ be the discriminant curve of the conic bundle $\textrm{pr}_1$. Then $\Delta _3$ is a cubic curve with at worst nodal singularities.

We claim that *A* does not fix points in $\Delta _3$. Indeed, suppose *A* fixes a point $p\in \Delta _3$. Let *F* be the fiber of the conic bundle $\textrm{pr}_1$ over *p* taken with reduced structure. Then *F* is *A*-invariant. Thus, if *p* is a smooth point of $\Delta _3$, then *F* has one singular point, and this point must be fixed by *A*. Similarly, if $p\in \textrm{Sing}(\Delta _3)$, then $\textrm{pr}_2(F)$ is a *A*-invariant line in $\mathbb {P}^2_{u,v,w}$, so Lemma 2.4 implies that *A* fixes a point in $\textrm{pr}_2(F)$, so that *A* fixes a point in *F*, since $\textrm{pr}_2$ induces an *A*-equivariant isomorphism $F\rightarrow \textrm{pr}_2(F)$. Thus, in both cases the group *A* fixes a point in *F* which is impossible by assumption. Hence, we conclude that *A* does not fix points in $\Delta _3$. If *X* is given by (4.6), then $\Delta _3$ is an irreducible cubic curve with one singular point, so *A* fixes the singular point of $\Delta _3$, which is impossible. Similarly, if *X* is given by (4.7), then $\Delta _3=L+C$, where *L* is a line, and *C* is a smooth conic. In this case, applying Lemma 2.4 to $\mathbb {P}^2_{x,y,z}$, we see that *A* fixes a point in p∈L, which we proved to be impossible. □

### Lemma 4.8

Let *X* be a smooth Fano 3-fold in Family N^o^3.2, and let *A* be a finite abelian subgroup in $\textrm{Aut}(X)$ that does not fix points on *X*. Then *X* is K-polystable.

### Proof

It is well-known that $-K_X$ is very ample and gives an *A*-equivariant embedding $X\hookrightarrow \mathbb {P}^9$, and quadrics in $\mathbb {P}^9$ containing the image cuts out a 4-fold $V\subset \mathbb {P}^9$ such that $V=\mathbb {P}\big (\mathcal {O}_{\mathbb {P}^1}(2)\oplus \mathcal {O}_{\mathbb {P}^1}(2)\oplus \mathcal {O}_{\mathbb {P}^1}(1)\oplus \mathcal {O}_{\mathbb {P}^1}(1)\big )$, and *X* is contained in the linear system |3M-4F|, where *M* is the tautological line bundle on *V*, and *F* is a fiber of the natural projection $V\rightarrow \mathbb {P}^1$. In the notation of [50, §2], we have $V=\mathbb {F}(2,2,1,1)$, and *X* is given by the following equation:

$$\begin{aligned}\begin{array}{c} \quad \quad \quad \alpha ^1_2(t_1,t_2)x_1^3+\alpha ^2_2(t_1,t_2)x_1^2x_2+\alpha ^1_1(t_1,t_2)x_1^2x_3+\alpha ^2_1(t_1,t_2)x_1^2x_4+ \\ +\alpha ^3_2(t_1,t_2)x_1x_2^2+\alpha ^3_1(t_1,t_2)x_1x_2x_3+\alpha ^4_1(t_1,t_2)x_1x_2x_4+\alpha ^1_0(t_1,t_2)x_1x_3^2+ \\ +\alpha ^2_0(t_1,t_2)x_1x_3x_4+\alpha ^3_0(t_1,t_2)x_1x_4^2+\alpha ^4_2(t_1,t_2)x_2^3+\alpha ^5_1(t_1,t_2)x_2^2x_3+ \\ +\alpha ^6_1(t_1,t_2)x_2^2x_4+\alpha ^4_0(t_1,t_2)x_2x_3^2+\alpha ^5_0(t_1,t_2)x_2x_3x_4+\alpha ^6_0(t_1,t_2)x_2x_4^2=0,\quad \quad \quad \end{array}\end{aligned}$$

where each $\alpha ^i_d(t_1,t_2)$ is a polynomial of degree *d*. This family is denoted by $T_{11}$ in [16].

Let $\pi :X\rightarrow \mathbb {P}^1_{t_1,t_2}$ be the morphism induced by the natural projection $V\rightarrow \mathbb {P}^1$, and let *S* be the surface in *X* that is given by $x_1=x_2=0$. Then π is *A*-equivariant, fibers of π are cubic surfaces in $\mathbb {P}^3$, the surface *S* is *A*-invariant, $S\cong \mathbb {P}^1\times \mathbb {P}^1$, the normal bundle of *S* in *X* is $\mathcal {O}_{\mathbb {P}^1\times \mathbb {P}^1}(-1,-1)$, and *S* contains a line on every fiber of π. Moreover, by Lemma 2.4 and Corollary 2.5, *A* acts without fixed points on $\mathbb {P}^1_{t_1,t_2}$.

Suppose that *X* is not K-polystable and let $\textbf{F}$ be an *A*-invariant divisor whose existence is asserted by Lemma 4.1, and let *Z* be its center on *X*. Then, as explained above, *Z* is an irreducible *A*-invariant curve, and $\pi (Z)=\mathbb {P}^1_{t_1,t_2}$, since *A* does not fix points in $\mathbb {P}^1_{t_1,t_2}$. Applying [7, Lemma 6.1] and [7, Lemma 6.2], we see that $\delta _p(X)>1$ for a general point p∈Z, which contradicts the existence of $\textbf{F}$. □

### Lemma 4.9

Let *X* be a smooth Fano 3-fold in Family N^o^3.5, and let *A* be a finite abelian subgroup in $\textrm{Aut}(X)$ that does not fix points on *X*. Then *X* is K-polystable.

### Proof

Set $S=\mathbb {P}^1\times \mathbb {P}^1$ and let C⊂S be a smooth curve of degree (5, 1) and construct *X* as follows. Consider the embedding $S\hookrightarrow \mathbb {P}^1\times \mathbb {P}^2$ given by $([u:v],[x:y])\mapsto ([u:v],[x^2:xy:y^2])$, and identify *S* and *C* with their images in $\mathbb {P}^1\times \mathbb {P}^2$ using this embedding. Then there exists a birational morphism $\pi :X\rightarrow \mathbb {P}^1\times \mathbb {P}^2$ that blows up *C*. Moreover, it follows from Lemma 2.13 that π is *A*-equivariant, both *C* and *S* are *A*-invariant, projection to the first factor $\textrm{pr}_1:\mathbb {P}^1\times \mathbb {P}^2 \rightarrow \mathbb {P}^1$ and projection to the second factor $\textrm{pr}_2:\mathbb {P}^1\times \mathbb {P}^2 \rightarrow \mathbb {P}^2$ are both *A*-equivariant. Note that *A* does not fix points in $\mathbb {P}^1\times \mathbb {P}^2$ by Theorem 2.1. On the other hand, applying Lemma 2.4 to $\mathbb {P}^2$ and the conic $\textrm{pr}_2(S)$, we see that *A* fixes a point in $\mathbb {P}^2$. Then *A* does not fix points in $\mathbb {P}^1$, so *X* is K-polystable by [4, Corollary 5.70]. □

### Lemma 4.10

Let *X* be a smooth Fano 3-fold in Family N^o^3.6, and let *A* be a finite abelian subgroup in $\textrm{Aut}(X)$ that does not fix points on *X*. Then *X* is K-polystable.

### Proof

Let us use the commutative diagram (2.1) together with all notations introduced in that diagram. Note that (2.1) is *A*-equivariant by Lemma 2.13. Moreover, *A* does not fix points in the left $\mathbb {P}^1$ in (2.1). Indeed, if *A* were to fix a point in the left $\mathbb {P}^1$ in (2.1), the fiber of $\phi _L$ over this point would be *A*-invariant, so its image in $\mathbb {P}^3$ would be an *A*-invariant plane, which would imply that *A* also fixes a point in $\mathbb {P}^3$ by Lemma 2.4, so *A* would fix a point in *X* by Theorem 2.1, contradicting our assumption.

Now, the K-polystability of *X* follows from [9]. Indeed, suppose that *X* is not K-polystable. Let $\textbf{F}$ be an *A*-invariant divisor whose existence is asserted by Lemma 4.1, and let *Z* be its center on *X*. Then *Z* is an irreducible *A*-invariant curve with $\varphi _L(Z)=\mathbb {P}^1$, since *A* does not fix points in the left $\mathbb {P}^1$ in (2.1). Now, applying [9, (★)] and [9, Corollary 2] to a general point p∈Z, we obtain

$$ 1\geqslant \delta (X)=\frac{A_X(\textbf{F})}{S_X(\textbf{F})}>1, $$

which is absurd. This completes the proof of the lemma. □

### Lemma 4.11

Let *X* be a smooth Fano 3-fold in Family N^o^3.10, and let *A* be a finite abelian subgroup in $\textrm{Aut}(X)$ that does not fix points on *X*. Then *X* is K-polystable.

### Proof

Suppose that the 3-fold *X* is not K-polystable. It follows from [4, §5.17] that there is a birational morphism $\pi :X\rightarrow Q$ such that *Q* is a smooth quadric 3-fold in $\mathbb {P}^4$, the morphism π is a blowup of two disjoint smooth conics $C_1=\{x=0,y=0,w^2+zt=0\}$ and $C_2=\{z=0,t=0,w^2+xy=0\}$, and either

4.8 $$\begin{aligned} Q=\big \{w^2+xy+zt+xt+xz=0\big \}, \end{aligned}$$

or

4.9 $$\begin{aligned} Q=\big \{w^2+xy+zt+a(xt+yz)+xz=0\big \} \end{aligned}$$

for some $a\in \mathbb {C}$ with $a\ne \pm 1$.

The description of the Mori cone $\overline{\textrm{NE}}(X)$ is given in [43, § III.3, p.80-p.81]. Using this description, we see that the morphism π is *A*-equivariant, and we have the following *A*-equivariant commutative diagram:where χ is a rational map given by [x:y:z:t:w]↦([x:y],[z:t]), and η is a (standard) conic bundle. Here, and below, we use ([*x* : *y*], [*z* : *t*]) as coordinates on $\mathbb {P}^1_{x,y}\times \mathbb {P}^1_{z,t}$, which may create a minor ambiguity.

Let Δ be the discriminant curve of the conic bundle η. Then Δ is an *A*-invariant curve of degree (2, 2), which has at worst nodal singularities. Moreover, if we are in case (4.8), then

$$ \Delta =\big \{x(t^2x+2txz-4tyz+xz^2)=0\big \}\subset \mathbb {P}^1_{x,y}\times \mathbb {P}^1_{z,t}. $$

Similarly, if we are in case (4.9), then

$$ \Delta =\big \{a^2t^2x^2+(2a^2-4)xyzt+2atzx^2+a^2y^2z^2+2ayz^2x+z^2x^2=0\big \}\subset \mathbb {P}^1_{x,y}\times \mathbb {P}^1_{z,t}, $$

If a≠0, this curve is irreducible with a node at ([0 : 1], [0 : 1]). If a=0, then $\Delta =\{zx(zx-4yt)=0\}$, and the curve Δ splits as a union of curves of degrees (0, 1), (1, 0), (1, 1). We claim that *A* does not fix points in Δ. Indeed, suppose that it does and let *F* be the fiber of the conic bundle η over an *A*-fixed point in Δ. Then *F* is singular. If *F* is reduced, then it has one singular point, and *A* must fix this point, which contradicts our assumption. If *F* is not reduced, then F=2ℓ for a smooth *A*-invariant curve ℓ with $\pi (\ell )$ an *A*-invariant line in *Q*. On the other hand, it follows from Lemma 2.4 that the action of the group *A* on the quadric *Q* is induced by a five-dimensional faithful representation of the group *A*, so the line $\pi (\ell )$ corresponds to a two-dimensional subrepresentation, which must split as a sum of two one-dimensional representations, since *A* is abelian. This shows that *A* fixes a point in $\pi (\ell )$, so *A* fixes a point in *X* by Theorem 2.1, which contradicts our assumption.

Hence, we see that *A* does not fix point in Δ. This implies that we are not in case (4.9). Thus, we are in case (4.8). In this case, we have Δ=L+Z, where *L* is a curve of degree (0, 1), and *Z* is an irreducible curve of degree (2, 1). Set $S=\pi _*(\eta ^*(L))$. Then *S* is a hyperplane section of the quadric *Q*, which is cut out on *Q* by the hyperplane $\{x=0\}$. One can check that *S* is a quadric cone with one singular point, so the vertex of *S* is fixed by *A*, which contradicts our assumption. □

### Lemma 4.12

Let *X* be a smooth Fano 3-fold in Family N^o^3.12, and let *A* be a finite abelian subgroup in $\textrm{Aut}(X)$ that does not fix points on *X*. Then *X* is K-polystable.

### Proof

There is a birational morphism $\pi :X\rightarrow \mathbb {P}^3$ which is the blowup of a line *L* and a twisted cubic *C*, which are disjoint. Let $f:V\rightarrow \mathbb {P}^3$ be the blowup of the curve *L*, and let $\widetilde{C}$ be the strict transform of the curve *C* on *V*. Then there exists a Sarkisov link:where *g* is a $\mathbb {P}^2$-bundle over $\mathbb {P}^1$. Moreover, the map *g* induces a finite morphism $\omega :\widetilde{C}\rightarrow \mathbb {P}^1$ of degree 3. Note that the description in [43, § III.3, p.83-p.84] implies that the blowup $X =\textrm{Bl}_{\widetilde{C}}(V) \rightarrow V$ is *A*-equivariant, and both *f* and *g* are *A*-equivariant. Furthermore, it follows from [23] that *X* is not K-polystable if and only if the triple cover $\omega :\widetilde{C}\rightarrow \mathbb {P}^1$ has a unique ramification point of ramification index 3. Thus, if *X* is not K-polystable, then the ramification point of index 3 of the finite morphism ω is fixed by *A*. Hence, if *X* is not K-polystable, then *A* fixes a point in *X* by Theorem 2.1, which contradicts our assumption. □

### Lemma 4.13

Let *X* be a smooth Fano 3-fold in Family N^o^3.13, and let *A* be a finite abelian subgroup in $\textrm{Aut}(X)$ that does not fix points on *X*. Then *X* is K-polystable.

### Proof

Suppose that *X* is not K-polystable. Then it follows from [4, Lemma 5.97], [4, Lemma 5.98] and [4, Proposition 5.99] that $\textrm{Aut}(X)\simeq \mathbb {G}_a\rtimes \mathfrak {S}_3$, which implies that either *A* is trivial, $A\simeq \mathbb {Z}/2\mathbb {Z}$, or $A\simeq \mathbb {Z}/3\mathbb {Z}$, which is impossible by Remark 2.3. □

### Lemma 4.14

Let *X* be a smooth Fano 3-fold in Family N^o^4.13, and let *A* be a finite abelian subgroup in $\textrm{Aut}(X)$ that does not fix points on *X*. Then *X* is K-polystable.

### Proof

In this case, there exists is a birational morphism $\pi :X\rightarrow \mathbb {P}^1_{x_0,x_1}\times \mathbb {P}^1_{y_0,y_1}\times \mathbb {P}^1_{z_0,z_1}$ which is the blowup of a smooth rational curve *C* such that *C* is a curve of degree (1, 1, 3). Suppose that *X* is not K-polystable. Then it follows from [4, § 5.22] that, up to a change of coordinates, we have

$$ C=\big \{x_0y_1-x_1y_0=x_0^3z_0+x_1^3z_1+x_0x_1^2z_0=0\big \}. $$

Moreover, it follows from the description of the extremal rays of the Mori cone $\overline{\textrm{NE}}(X)$ given in [43, § III.3, p.84-p.85] that π and the projection $X\rightarrow \mathbb {P}^1_{z_0,z_1}$ given by $([x_0,x_1],[y_0,y_1],[z_0,z_1])\mapsto [z_0,z_1]$ are both *A*-equivariant, so *C* is *A*-invariant. On the other hand, the projection $X\rightarrow \mathbb {P}^1_{z_0,z_1}$ induces a triple cover $C\rightarrow \mathbb {P}_{z_0,z_1}$, which has a unique ramification point with ramification index 3, so this point must be fixed by *A*, which contradicts out assumption. □

## Examples of Smooth Fano 3-Folds Not Satisfying Condition $\mathbf {(A)}$

What is all this games stuff, anyway? In a way, it’s easy enough to see what happens to winners who stay on Earth. They get all the juicy jobs; they become governors and such. ——————————————————————– *The World of Null-A*, A. E. van Vogt, 1948

In this section, we provide examples of smooth Fano 3-folds that do not satisfy Condition $\mathbf {(A)}$.

### K-Unstable Smooth Fano 3-Folds Not Satisfying Condition $\mathbf {(A)}$.

To complete Main Theorem, in this subsection we will give eight exceptions of non K-polystable smooth Fano threefolds which do not satisfy Condition $\mathbf {(A)}$ as mentioned in Main Theorem.

#### Example 5.1

(Families N^o^3.28 and N^o^4.10) Let $X=\mathbb {P}^1\times S$, where *S* is a blow up of $\mathbb {P}^2$ in one or two points. Then $\textrm{Aut}(X)$ contains a subgroup isomorphic to $(\mathbb {Z}/2\mathbb {Z})^2$ that acts trivially on the second factor, and does not fix points in *X*.

#### Example 5.2

(Family N^o^2.23) Let *X* be a smooth Fano 3-fold in Family N^o^2.23. Then *X* is the blowup of a smooth quadric 3-fold in $Q\subset \mathbb {P}^4$ along a smooth quartic elliptic curve $C_4$. Let *S* be the unique hyperplane section of *Q* that contains $C_4$. If *S* is singular, then *X* satisfiess Condition $\mathbf {(A)}$ by Remark 3.9. On the other hand, if *S* is smooth, then *X* does not satisfy Condition $\mathbf {(A)}$. Indeed, suppose that *S* is smooth. Then it follows from [49, Proposition 2.1] that we can choose coordinates $x_1,x_2,x_3,x_4,x_5$ on $\mathbb {P}^4$ such that $Q=\{x_1^2+x_2^2+x_3^2+x_4^2+x_5^2=0\}$, and the curve $C_4$ is given by the following equations:

$$ x_5=\sum _{i=1}^{5}x_i^2=\sum _{i=1}^{5}\lambda _ix_i^2=0 $$

for some $\lambda _1,\ldots ,\lambda _5\in \mathbb {C}$. Let *A* be the abelian subgroup in $\textrm{Aut}(Q)$ consisting of projective transformations that change signs of coordinates $x_1,\ldots ,x_5$. Then $A\simeq (\mathbb {Z}/2\mathbb {Z})^4$, the group *A* does not fix points in *Q*, and the curve $C_4$ is *A*-invariant, so the action of the group *A* lifts to *X*, and *A* does not fix points in *X*.

#### Example 5.3

(Family N^o^3.31) Let *Q* be a quadric cone in $\mathbb {P}^4$ with one singular point, and let f:X→Q be the blowup of its vertex. Then choosing appropriate coordinates on $\mathbb {P}^4$, we may assume that $Q=\{x_1^2+x_2^2+x_3^2+x_4^2=0\}$. Let *A* be the abelian subgroup in $\textrm{Aut}(Q)$ consisting of projective transformations that change signs of coordinates $x_1,\ldots ,x_5$. Then $A\simeq (\mathbb {Z}/2\mathbb {Z})^4$, the only *A*-fixed point in *Q* is its vertex [0 : 0 : 0 : 0 : 1], and the action of the group *A* lifts to *X*. Note that there exists *A*-equivariant commutative diagramwhere *S* is the quadric surface in $\mathbb {P}^3$ given by $x_1^2+x_2^2+x_3^2+x_4^2=0$ equipped with the same action of the group *A*, π is a $\mathbb {P}^1$-bundle, and χ is given by $[x_1:x_2:x_3:x_4:x_5]\mapsto [x_1:x_2:x_3:x_4]$. Since *A* does not fix points in *S*, we see that *A* does not fix points in *X* either.

#### Example 5.4

(Family N^o^4.8) Let *X* be the blowup of $\mathbb {P}^1_{x_1,y_1}\times \mathbb {P}^1_{x_2,y_2}\times \mathbb {P}^1_{x_3,y_3}$ along a smooth curve *C* of degree (0, 1, 1). Then, changing coordinates if necessary, we may assume that $C=\{y_1=0,x_2y_3-x_3y_2=0\}$. Let *A* be the subgroup in $\textrm{Aut}(\mathbb {P}^1_{x_1,y_1}\times \mathbb {P}^1_{x_2,y_2}\times \mathbb {P}^1_{x_3,y_3})$ generated by the following transformations:

$$\begin{aligned} ([x_1:y_1],[x_2:y_2],[x_3:y_3])&\mapsto ([x_1:y_1],[x_2:-y_2],[x_3:-y_3]),\\ ([x_1:y_1],[x_2:y_2],[x_3:y_3])&\mapsto ([x_1:y_1],[y_2:x_2],[y_3:x_3]). \end{aligned}$$

Then $A\simeq (\mathbb {Z}/2\mathbb {Z})^2$, it does not fix points in $\mathbb {P}^1_{x_1,y_1}\times \mathbb {P}^1_{x_2,y_2}\times \mathbb {P}^1_{x_3,y_3}$, and it leaves the curve *C* invariant. Hence, the action of the group *A* lifts to *X*, and *A* does not fix points in *X*.

#### Example 5.5

(Families N^o^2.33, N^o^4.12, N^o^5.2) Let *A* be the subgroup in $\textrm{Aut}(\mathbb {P}^3)$ generated by

$$\begin{aligned} [x_1:x_2:x_3:x_4]&\mapsto [x_1:-x_2:x_3:-x_4],\\ [x_1:x_2:x_3:x_4]&\mapsto [x_2:x_1:x_4:x_3]. \end{aligned}$$

Then $A\simeq (\mathbb {Z}/2\mathbb {Z})^2$ and it does not fix points in $\mathbb {P}^3$, and *A* leaves invariant the lines $L_1=\{x_1=x_2=0\}$ and $L_2=\{x_3=x_4=0\}$. Blowing up $\mathbb {P}^3$ along $L_1$, we obtain the exceptional case (6) in the Main Theorem. Blowing up the points [0 : 0 : 1 : 0] and [0 : 0 : 0 : 1], and then blowing up the strict transform of the line $L_1$, we obtain the exceptional case (7). Finally, blowing up [0 : 0 : 1 : 0] and [0 : 0 : 0 : 1], and then blowing up the strict transforms of the lines $L_1$ and $L_2$, we obtain the exceptional case (8). In each case, the action of the group *A* lifts to the resulting Fano 3-fold, and *A* does not fix points in it.

### K-Polystable Smooth Fano 3-Folds Not Satisfying Condition $\mathbf {(A)}$.

#### Example 5.6

Let $X_3$ be the cubic 3-fold $\{x_1x_2x_3+(x_1+x_2+x_3)x_4x_5+2025(x_4^3+x_5^4)=0\}\subset \mathbb {P}^4$, and let *A* be the subgroup in $\textrm{Aut}(X_3)$ generated by the following transformations:

$$\begin{aligned} [x_1:x_2:x_3:x_4:x_5]&\rightarrow [x_2:x_3:x_1:x_4:x_5],\\ [x_1:x_2:x_3:x_4:x_5]&\rightarrow [x_1:x_2:x_3:\zeta _3x_4:\zeta _3^2x_5], \end{aligned}$$

where $\zeta _3$ is a primitive cube root of unity. Then $X_3$ has three ordinary double point singularities (nodes) at [1 : 0 : 0 : 0 : 0], [0 : 1 : 0 : 0 : 0], [0 : 0 : 1 : 0 : 0], $A\simeq (\mathbb {Z}/3\mathbb {Z})^2$, and the group *A* does not fix points in $X_3$. Moreover, it follows from [20, Section 4] that we have the following *A*-equivariant commutative diagram:where φ is the blowup of the nodes of $X_3$, α is the small birational morphism that contracts the strict transform of three lines in $X_{3}$ that contain two nodes of $X_3$, ζ is a pseudoautomorphism that flops curves contracted by α, *V* is a Fano 3-fold with three nodes such that $\textrm{Pic}(V)\simeq \mathbb {Z}$ and $(-K_V)^3=18$, β is a small birational morphism, π is a fibration into del Pezzo surfaces of degree 6, and χ is a rational map that is given by $[x_1:x_2:x_3:x_4:x_5]\mapsto [x_4:x_5]$. Moreover, the abelian group *A* does not fix points in *V*. Furthermore, by Namikawa [45], *V* is smoothable. Infinitesimal deformations are parametrized by the complex vector space $H^1(V,T_V)$ on which *A* acts linearly. As *A* is abelian, this representation splits and we can choose an *A*-equivariant smoothing by [51], cf. [25, Lemma 3.7]. Let *X* be a general such smoothing. Then *A* does not fix points on *X* as well, and *X* is a smooth Fano 3-fold belonging to Family N^o^1.9 which does not satisfy Condition $\mathbf {(A)}$.

#### Example 5.7

([4, Example 4.25]) Consider the smooth cubic 3-fold $V=\{x_1^3+x_2^3+x_3^3+x_4^3+x_5^3=0\}\subset \mathbb {P}^4$, let *C* be the smooth cubic curve in *V* that is cut out by the plane $\{x_1=x_2=0\}$, and let $\pi :X\rightarrow V$ be the blowup of the curve *C*, where $x_1$, $x_2$, $x_3$, $x_4$, $x_5$ are coordinates on $\mathbb {P}^4$. Then *X* is a smooth Fano 3-fold in Family N^o^2.5. Let *A* be the abelian subgroup in $\textrm{Aut}(V)$ that is generated by transformations

$$ [x_1:x_2:x_3:x_4:x_5]\mapsto [\zeta _3^{a_1}x_1:\zeta _3^{a_2}x_2:\zeta _3^{a_3}x_3:\zeta _3^{a_4}x_4:x_5], $$

where $\zeta _3$ is a primitive cube root of unity, and each $a_i\in \{0,1,2\}$. Then $A\simeq (\mathbb {Z}/3\mathbb {Z})^4$, and *A* does not fix points in *V*. Moreover, since *C* is *A*-invariant, the action of the group *A* lifts to *X*, so we can identify *A* with a subgroup in $\textrm{Aut}(X)$. Then *A* does not fix points in *X* by Theorem 2.1,

#### Example 5.8

In $\mathbb {P}^5$, we consider the following complete intersection of two quadrics:

$$ V=\Big \{\sum _{i=1}^6 x_i^2 = \sum _{i=1}^6 a_i x_i^2 =0\Big \}\subset \mathbb {P}^5, $$

where $a_1,a_2,a_3,a_4,a_5,a_6$ are general complex numbers. Let $C=\{x_1=x_2=0\}\cap V$, and let *A* be the subgroup in *V* generated by the following involutions:

$$\begin{aligned} [x_1:x_2:x_3:x_4:x_5:x_6]&\mapsto [-x_1:x_2:x_3:x_4:x_5:x_6],\\ [x_1:x_2:x_3:x_4:x_5:x_6]&\mapsto [x_1:-x_2:x_3:x_4:x_5:x_6],\\ [x_1:x_2:x_3:x_4:x_5:x_6]&\mapsto [x_1:x_2:-x_3:x_4:x_5:x_6],\\ [x_1:x_2:x_3:x_4:x_5:x_6]&\mapsto [x_1:x_2:x_3:-x_4:x_5:x_6],\\ [x_1:x_2:x_3:x_4:x_5:x_6]&\mapsto [x_1:x_2:x_3:x_4:-x_5:x_6]. \end{aligned}$$

Then *V* is smooth, *C* is smooth and *A*-invariant, $A\simeq (\mathbb {Z}/2\mathbb {Z})^5$, the group *A* does not fix points in *V*. Now, let $\pi :X\rightarrow V$ be the blowup of the curve *C*. Then *X* is a smooth Fano 3-fold in Family N^o^2.10 on which the lifted action of *A* does not fix points.

#### Example 5.9

In [15, Section 2.1], there is an explicit example of a smooth sextic curves $C_6\subset \mathbb {P}^3$ of genus 3 such that $\textrm{Aut}(\mathbb {P}^3,C_6)$ contains a subgroup $G\simeq \mathfrak {S}_4$. The generators of the subgroup *G* are listed in the proof of [15, Lemma 10]. Let *A* be the unique subgroup in *G* that is isomorphic to $(\mathbb {Z}/2\mathbb {Z})^2$. Then *A* does not fix points in $\mathbb {P}^3$. By blowing up $\mathbb {P}^3$ along $C_6$ we obtain a smooth Fano 3-fold *X* in Family N^o^2.12 with $\textrm{Aut}(X)$ containing a subgroup isomorphic to $(\mathbb {Z}/2\mathbb {Z})^2$ that does not fix points in *X*.

#### Example 5.10

Let $C=\{x_1^2+x_2^2+x_3^2=0,x_4=0,x_5=0,x_6=0\}\subset \mathbb {P}^5$, let *V* be the complete intersection of two quadrics in $\mathbb {P}^5$ that is given by

$$ {\left\{ \begin{array}{ll} & x_1^2+x_2^2+x_3^2+x_4^2+x_5^2+x_6^2=0, \\ &  a x_1x_4+b x_2x_5+ c x_3x_6=0, \end{array}\right. } $$

with abc≠0, and let *A* be the subgroup in $\textrm{Aut}(V)$ generated by the following involutions:

$$\begin{aligned} [x_1:x_2:x_3:x_4:x_5:x_6]&\mapsto [-x_1:x_2:x_3:-x_4:x_5:x_6],\\ [x_1:x_2:x_3:x_4:x_5:x_6]&\mapsto [x_1:-x_2:x_3:x_4:-x_5:x_6],\\ [x_1:x_2:x_3:x_4:x_5:x_6]&\mapsto [x_2:x_1:x_3:x_5:x_4:x_6]. \end{aligned}$$

Then C⊂V, both *C* and *V* are smooth, $A\simeq (\mathbb {Z}/2\mathbb {Z})^3$, *A* does not fix points in *V*, and *C* is *A*-invariant. Let $\pi :X\rightarrow V$ be the blowup of the conic *C*. Then *X* is a smooth Fano 3-fold in Family N^o^2.16, the action of the group *A* lifts to *X*, and *A* does not fix points in *X*.

#### Example 5.11

Let *Q* be the quadric 3-fold in $\mathbb {P}^4$ given by $4x_2x_4-x_1x_5-3x_3^2=0$, and let *C* be the twisted quartic curve in $\mathbb {P}^4$ that is given in the parametric form as $[u:v]\mapsto [u^4:u^3v:u^2v^2:uv^3:v^4]$, where $[u:v]\in \mathbb {P}^1$. Then *Q* is smooth and contains *C*. Let $\pi :X\rightarrow Q$ be the blowup of the curve *C*. Then *X* is a smooth Fano 3-fold in Family N^o^2.21, and it follows from [4, § 5.9] or from [41] that the group $\textrm{Aut}(X)$ contains an involution σ creating the following commutative diagram:where τ is a birational involution of the quadric 3-fold *Q* that is given by

$$\begin{aligned} [x_1:x_2:x_3:x_4:x_5]\mapsto &   \big [4(x_1x_3-x_2^2): 2(x_1x_4-x_2x_3):(x_1x_5-x_3^2)\\  &   :2(x_2x_5-x_3x_4):4(x_3x_5-x_4^2)\big ]. \end{aligned}$$

Now, we let *A* be the subgroup in $\textrm{Aut}(X)$ generated by σ and the lifts of the following two transformations:

$$\begin{aligned} [x_1:x_2:x_3:x_4:x_5]&\mapsto [x_5:x_4:x_3:x_2:x_1],\\ [x_1:x_2:x_3:x_4:x_5]&\mapsto [x_1:-x_2:x_3:-x_4:x_5]. \end{aligned}$$

Then $A\simeq (\mathbb {Z}/2\mathbb {Z})^3$, and *A* does not fix points in *X*.

#### Example 5.12

Let *X* be the divisor in $\mathbb {P}^2_{x,y,z}\times \mathbb {P}^2_{u,v,w}$ that is given by the following equation:

$$ xu^2+yv^2+zw^2+\mu \big (xvw+yuw+zuv\big )=0, $$

where $\mu \in \mathbb {C}$ such that $\mu ^3\ne -1$. Then *X* is a smooth Fano 3-fold in Family N^o^2.24, and every K-polystable smooth member in Family N^o^2.24 can be obtained in this way [4, § 4.7]. Let *A* be the subgroup in $\textrm{Aut}(X)$ that is generated by the following transformations:

$$\begin{aligned} \big ([x:y:z],[u:v:w]\big )&\mapsto \big ([y:z:x],[v:w:u)\big ]\big ),\\ \big ([x:y:z],[u:v:w]\big )&\mapsto \big ([\zeta _3 x:\zeta _3^2 y:z],[\zeta _3 u:\zeta _3^2 v:w]\big ). \end{aligned}$$

Then $A\simeq (\mathbb {Z}/3\mathbb {Z})^2$ and *A* does not fix points in *X*.

#### Example 5.13

Let $V=\mathbb {P}\big (\mathcal {O}_{\mathbb {P}^1}(2)\oplus \mathcal {O}_{\mathbb {P}^1}(2)\oplus \mathcal {O}_{\mathbb {P}^1}(1)\oplus \mathcal {O}_{\mathbb {P}^1}(1)\big )$, let *M* be the tautological line bundle on *V*, and let *F* be a fiber of the natural projection $V\rightarrow \mathbb {P}^1$. In the notations used in [50, §2], let *X* be the 3-fold in the linear system |3M-4F| that is given by the following equation:

$$\begin{aligned}\begin{array}{c} \quad \quad \quad \quad \quad x_1x_3x_4+3x_2(x_3^2+x_4^2)+1007t_1t_2x_1^3-301t_1t_2x_1x_2^2+ \\ +(t_1^2+t_2^2)(71x_1^2x_2-54x_2^3)+1111x_1^2(t_2x_3+t_1x_4)-99x_2^2(t_2x_3+t_1x_4)=0. \quad \quad \quad \quad \quad \quad \end{array}\end{aligned}$$

Then *X* is a smooth Fano 3-fold in the family N^o^3.2. This family is denoted by $T_{11}$ in [16]. Note that the natural projection $V\rightarrow \mathbb {P}^1$ induces a morphism $\pi :X\rightarrow \mathbb {P}^1_{t_1,t_2}$ whose general fiber is a smooth cubic surface in $\mathbb {P}^3$. Let *A* be the subgroup in $\textrm{Aut}(V)$ that is generated by the following transformations:

$$\begin{aligned} (t_1,t_2;x_1,x_2,x_3,x_4)&\mapsto (t_2,t_1;x_1,x_2,x_4,x_3),\\ (t_1,t_2;x_1,x_2,x_3,x_4)&\mapsto (t_1,-t_2;x_1,-x_2,x_3,-x_4). \end{aligned}$$

Then $A\simeq (\mathbb {Z}/2\mathbb {Z})^2$, the 3-fold *X* is *A*-invariant, the group *A* acts faithfully on *X*, and the morphism π is *A*-equivariant. Moreover, the group *A* does not fix points in $\mathbb {P}^1_{t_1,t_2}$, so that it does not fix points in *X*.

#### Example 5.14

Let $S=\mathbb {P}^1_{u,v}\times \mathbb {P}^1_{x,y}$, and let *A* be the subgroup in $\textrm{Aut}(S)$ generated by

$$\begin{aligned} ([u:v],([x:y])&\mapsto ([v:u],[y:x]),\\ ([u:v],([x:y])&\mapsto ([-u:v],[-x:y]). \end{aligned}$$

Then it follows from [4, Lemma A.54] that *S* contains an *A*-invariant smooth curve *C* of degree (5, 1). Now, we consider the *A*-equivariant embedding $S\hookrightarrow \mathbb {P}^1\times \mathbb {P}^2$ given by

$$ \big ([u:v],[x:y]\big )\mapsto \big ([u:v],[x^2:xy:y^2]\big ). $$

We identify *S* and *C* with their images in $\mathbb {P}^1\times \mathbb {P}^2$, and we identify *A* with a subgroup in $\textrm{Aut}(\mathbb {P}^1\times \mathbb {P}^2)$. Then *A* does not fix points in $\mathbb {P}^1\times \mathbb {P}^2$. Let $\pi :X\rightarrow \mathbb {P}^1\times \mathbb {P}^2$ be the blow up along *C*. Then *X* is a smooth Fano 3-fold in Family N^o^ 3.5, the action of the group *A* lifts to *X*, and *A* does not fix points in *X*.

#### Example 5.15

Let *L* be the line $\{x_1-x_3=x_2-x_4=0\}\subset \mathbb {P}^3$, let *C* be the curve

$$ \big \{x_1^2+x_2^2+a(x_3^2+x_4^2)=0, a(x_1^2-x_2^2)+x_3^2-x_4^2=0\big \}. $$

with a≠0,1. Then $L\cap C=\varnothing$, and *C* is a smooth elliptic curve. Let $\pi :X\rightarrow \mathbb {P}^3$ be the blow up along *L* and *C*. Then *X* is a smooth Fano 3-fold in Family N^o^3.6. Let *A* be the subgroup in $\textrm{Aut}(\mathbb {P}^3)$ generated by

$$\begin{aligned} [x_1:x_2:x_3:x_4]&\mapsto [x_2:x_1:x_4:x_3],\\ [x_1:x_2:x_3:x_4]&\mapsto [x_1:-x_2:x_3:-x_4]. \end{aligned}$$

Then $A\simeq (\mathbb {Z}/2\mathbb {Z})^2$, and both curves *L* and *C* are *A*-invariant, so the action of the group *A* lifts to *X*. Moreover, the group *A* does not fix points in $\mathbb {P}^3$, so that it does not fix points in *X*.

#### Example 5.16

Let *A* be the subgroup in $\textrm{Aut}(\mathbb {P}^2\times \mathbb {P}^2)$ that is generated by the following transformations:

$$\begin{aligned} ([x_1:x_2:x_3],[y_1:y_2:y_3])&\mapsto ([x_2:x_3:x_1],[y_2:y_3:y_1]),\\ ([x_1:x_2:x_3],[y_1:y_2:y_3])&\mapsto ([\zeta _3^2x_1:\zeta _3x_2:x_3],[\zeta _3y_1:\zeta _3^2y_2:y_3]), \end{aligned}$$

where $\zeta _3$ is a primitive cube root of unity. Then $A\simeq (\mathbb {Z}/3\mathbb {Z})^2$, and *A* acts on $\mathbb {P}^2\times \mathbb {P}^2$ without fixed points. Let *W*, $W^\prime$, $W^{\prime \prime }$ be divisors of degree (1, 1) in $\mathbb {P}^2\times \mathbb {P}^2$ that are given by

$$\begin{aligned}\begin{gathered} x_1y_1+x_2y_2+x_3y_3=0, \\ \zeta _3x_1y_1+\zeta _3^2x_2y_2+x_3y_3=0, \\ x_1y_2+x_2y_3+x_3y_1=0, \end{gathered}\end{aligned}$$

respectively. Then they are smooth and *A*-invariant. Set $C=W\cap W^\prime \cap W^{\prime \prime }$. Then *C* is a smooth elliptic curve. Let $\pi :X\rightarrow W$ be the blowup of the curve *C*. Then *X* is a smooth Fano 3-fold in Family N^o^3.7, and the action of the group *A* lifts to *X*, and *A* does not fix points in *X*.

#### Example 5.17

Let *Q* be the smooth quadric 3-fold in $\mathbb {P}^4$ that is given by $x_1^2+x_2^2+x_3^2+x_4^2+x_5^2=0$, let $\Pi _1=\{x_1=x_2=0\}$ and $\Pi _2=\{x_2=x_4=0\}$, and let *A* be the subgroup in $\textrm{Aut}(Q)$ generated by

$$\begin{aligned} [x_1:x_2:x_3:x_4:x_5]&\mapsto [-x_1:x_2:x_3:x_4:x_5],\\ [x_1:x_2:x_3:x_4:x_5]&\mapsto [x_1:-x_2:x_3:x_4:x_5],\\ [x_1:x_2:x_3:x_4:x_5]&\mapsto [x_1:x_2:-x_3:x_4:x_5],\\ [x_1:x_2:x_3:x_4:x_5]&\mapsto [x_1:x_2:x_3:-x_4:x_5]. \end{aligned}$$

Then $A\simeq (\mathbb {Z}/2\mathbb {Z})^4$, and *A* does not have fix points on *Q*, planes $\Pi _1$ and $\Pi _2$ are *A*-invariant. Let $\pi :X\rightarrow Q$ be the blowup of the disjoint conics $Q\cap \Pi _1$ and $Q\cap \Pi _2$. Then *X* is a smooth Fano 3-fold in Family N^o^3.10, the action of the group *A* lifts to *X*, and *A* does not fix points in *X*.

#### Example 5.18

Let *L* be the line $\{x_1=x_4=0\}\subset \mathbb {P}^3$, let *C* be the twisted cubic $\varphi (\mathbb {P}^1)$ for $\varphi :\mathbb {P}^1\rightarrow \mathbb {P}^3$ given by $[u:v]\mapsto [v^3:uv^2:u^2v:u^3]$. Then $L\cap C=\varnothing$. Let $\pi :X\rightarrow \mathbb {P}^3$ be the blowup along *L* and *C*. Then *X* is a smooth Fano 3-fold in Family N^o^3.12. Let *A* be the subgroup in $\textrm{Aut}(\mathbb {P}^3)$ generated by

$$\begin{aligned} [x_1:x_2:x_3:x_4]&\mapsto [x_4:x_3:x_2:x_1],\\ [x_1:x_2:x_3:x_4]&\mapsto [x_1:-x_2:x_3:-x_4]. \end{aligned}$$

Then $A\simeq (\mathbb {Z}/2\mathbb {Z})^2$, both curves *L* and *C* are *A*-invariant, so the action of the group *A* lifts to *X*. Moreover, the group *A* does not fix points in $\mathbb {P}^3$, hence it does not fix points in *X*.

#### Example 5.19

In $\mathbb {P}^2_{x_1,x_2,x_3}\times \mathbb {P}^2_{y_1,y_2,y_3}\times \mathbb {P}^2_{z_1,z_2,z_3}$, we let

$$ X=\big \{x_1y_1+x_2y_2+x_3y_3=0,x_1z_1+x_2z_2+x_3z_3=0,y_1z_1+y_2z_2+y_3z_3=0\big \}. $$

Then *X* is a smooth Fano 3-fold in Family N^o^3.13, and $\textrm{Aut}(X)\simeq \textrm{PGL}_2(\mathbb {C})\times \mathfrak {S}_3$ by [4, Remark 5.100]. Let *A* be the subgroup in $\textrm{Aut}(X)$ generated by

$$\begin{aligned} \big ([x_1:x_2:x_3],[y_1:y_2:y_3],[z_1:z_2:z_3]\big )&\mapsto \big ([-x_1:x_2:x_3],[-y_1:y_2:y_3],[-z_1:z_2:z_3]\big ),\\ \big ([x_1:x_2:x_3],[y_1:y_2:y_3],[z_1:z_2:z_3]\big )&\mapsto \big ([x_1:-x_2:x_3],[y_1:-y_2:y_3],[z_1:-z_2:z_3]\big ),\\ \big ([x_1:x_2:x_3],[y_1:y_2:y_3],[z_1:z_2:z_3]\big )&\mapsto \big ([y_1:y_2:y_3],[x_1:x_2:x_3],[z_1:z_2:z_3]\big ). \end{aligned}$$

Then $A\simeq (\mathbb {Z}/2\mathbb {Z})^3$, and *A* does not fix points in *X*.

#### Example 5.20

([4, § 5.22]) Let $V=\mathbb {P}^1_{x_1,x_2}\times \mathbb {P}^1_{y_1,y_2}\times \mathbb {P}^1_{z_1,z_2}$, and let *A* be the subgroup of ${\text {Aut}}(V)$ generated by the transformations

$$\begin{aligned} \big ([x_1 :x_2],[y_1 :y_2],[z_1 :z_2]\big )&\mapsto \big ([x_1 :-x_2],[y_1 :-y_2],[z_1 :-z_2]\big ),\\ \big ([x_1 :x_2],[y_1 :y_2],[z_1 :z_2]\big )&\mapsto \big ([x_2 :x_1],[y_2 :y_1],[z_2 :z_1]\big ), \\ \big ([x_1 :x_2],[y_1 :y_2],[z_1 :z_2]\big )&\mapsto \big ([y_1 :y_2],[x_1 :x_2],[z_1 :z_2]\big ). \end{aligned}$$

Then $A\simeq (\mathbb {Z}/2\mathbb {Z})^3$, and *A* does not fix points in *V*. Let *C* be the curve of degree (1, 1, 3) in *V* given by

$$ x_1y_2 - x_2y_1 = x_1^3z_1 + x_2^3z_2 + \lambda (x_1x_2^2z_1 + x_1^2x_2z_2) = 0, $$

where $\lambda \in \mathbb {C}\setminus \{\pm 1,\pm 3\}$. Then *C* is smooth and *A*-invariant. Let $\pi :X \rightarrow V$ be the blowup of *V* along *C*. Then *X* is a smooth Fano 3-fold in Family N^o^4.13, and it follows from [4, § 5.22] that every K-polystable smooth Fano 3-fold in Family N^o^4.13 can be obtained in this way. Since *C* is *A*-invariant, the action of the group *A* lifts to *X*, and *S* does not fix points in *X*.

## Data Availability

Not Applicable.

## Footnotes

1 $\textrm{lct}_{S\cap Z}(S;D)$ is not defined anywhere in the literature!
